# Supplementary material for: Nanopore- and AI-empowered microbial viability inference
Source: Gigascience. 2025 Sep 3;14:giaf100. doi: 10.1093/gigascience/giaf100 (PMC12405693; doi:10.1093/gigascience/giaf100)
Supplement: giaf100_Supplemental_File [file giaf100_supplemental_file.pdf]

Supporting information

Table S1. UV-killed *E. coli* viability inferences of deep neural network and logistic regression models Test dataset performance metrics of residual neural networks (“ResNet”), transformer architectures, and logistic regression models, trained on various data modalities (nanopore squiggle “Signal” or basecalled DNA “Nucleotide” sequence) at various signal chunk sizes (“Length”), and on sequencing read length or translocation speed (“Trans speed”).

Model Architecture	Data Modality	Length	Accuracy	F1	Precision	Sensitivity	Specificity	AUROC	AUPR
ResNet1	Signal	10K	0.83	0.84	0.78	0.91	0.74	0.90	0.87
ResNet2	Signal	10K	0.83	0.85	0.77	0.94	0.72	0.89	0.86
ResNet3	Signal	10K	0.81	0.82	0.76	0.89	0.72	0.87	0.83
Transformer	Signal	10K	0.79	0.82	0.73	0.92	0.67	0.86	0.82
ResNet1	Nucleotide (A,C,G,T)	800	0.51	0.51	0.51	0.52	0.50	0.52	0.53
ResNet1	Nucleotide (A,C,G,T, M)	800	0.51	0.50	0.51	0.50	0.52	0.51	0.51
ResNet1	Signal	1K	0.58	0.65	0.55	0.80	0.35	0.61	0.58
ResNet1	Signal	5K	0.71	0.76	0.66	0.89	0.54	0.78	0.73
ResNet1	Signal	7K	0.77	0.79	0.72	0.88	0.65	0.83	0.79
ResNet1	Signal	12K	0.84	0.85	0.77	0.97	0.71	0.91	0.88
ResNet1	Signal	20K	0.89	0.90	0.85	0.95	0.83	0.94	0.91
Regression	Read length	NA	0.50	0.00	0.00	0.00	1.00	0.56	0.56
Regression	Trans speed	NA	0.59	0.55	0.61	0.50	0.68	0.63	0.59

Table S2. Sequencing read-level viability inferences of UV ResNet1

Performance metrics across sequencing reads of the ResNet1 model trained on UV-killed *E. coli* for the *E. coli* test dataset, and two biological replicates (BR1 and BR2) of UV-killed and viable *Chlamydia suis*. The number of total reads is the number of sequencing reads after processing of the nanopore sequencing data by Porechop; the number of genus-classified reads is the number of sequencing reads that map to the *Escherichia* or *Chlamydia* genus, respectively, using Kraken2 (Materials and Methods).

Metric	*E. coli* test dataset	*C. suis* BR1	*C. suis* BR2
**#total reads**	140,268	63,935	76,418
**#genus-classified reads**	131,378	36,981	40,945
**Accuracy**	0.96	0.93	0.93
**F1 Score**	0.96	0.87	0.90
**Sensitivity**	0.94	0.95	0.96
**Specificity**	0.97	0.92	0.91
**Precision**	0.98	0.80	0.85
**AUPR**	0.99	0.94	0.96
**AUROC**	0.99	0.97	0.97

Table S3. Cultivation, viability PCR (vPCR) and nanopore sequencing metrics of UV-killed *Chlamydia suis*. Cultivation titer in number of Inclusion Forming Units (IFUs) per mL; PMA-untreated vPCR reflecting total *Chlamydia* content, PMA-treated vPCR reflecting viable *Chlamydia* content, and Δlog10 of the PMA-treated copy number divided by the PMA-untreated copy-number reflecting overall viability; total number of nanopore sequencing reads after Porechop-processing, and number of *Chlamydia*-classified reads using Kraken2 (Materials and Methods), of two biological replicates (BR1 and BR2) of viable and dead *C. suis*.

Condition	Titer [IFU/mL]	PMA-untreated vPCR [copy number per mL]	PMA-treated vPCR [copy number per mL]	Δlog10 of viability ratio	#total reads	#genus-classified reads
**viable BR1**	$8.48e^{7}$	$9.63e^{7}$	$1.47e^{7}$	0.82	37,439	28,210
**dead BR1**	0	$7.16e^{5}$	$2.23e^{4}$	1.51	26,496	8,771
**viable BR2**	$6.23e^{7}$	$9.15e^{7}$	$1.24e^{7}$	0.81	34,444	26,643

**dead BR2**	0	$1.03e^{6}$	$9.81e^{3}$	2.02	41,974	14,302

Table S4. Sequencing read-level viability inferences of antibiotic exposure ResNet1

Performance metrics across sequencing reads of the ResNet1 model trained on antibiotic-exposed *E. coli* for the *E. coli* test dataset, and a heldout biological replicates (BR). The number of *E. coli* reads is the number of sequencing reads after processing of the nanopore sequencing data by Porechop and mapping to the *Escherichia* genus using Kraken2 (Materials and Methods).

Metric	Test dataset	BR
**#*E. coli* reads**	49,781	119,930
**Accuracy**	0.73	0.68
**F1**	0.82	0.78
**Sensitivity**	0.71	0.66
**Specificity**	0.87	0.80
**Precision**	0.98	0.95
**AUPR**	0.98	0.95
**AUROC**	0.87	0.80

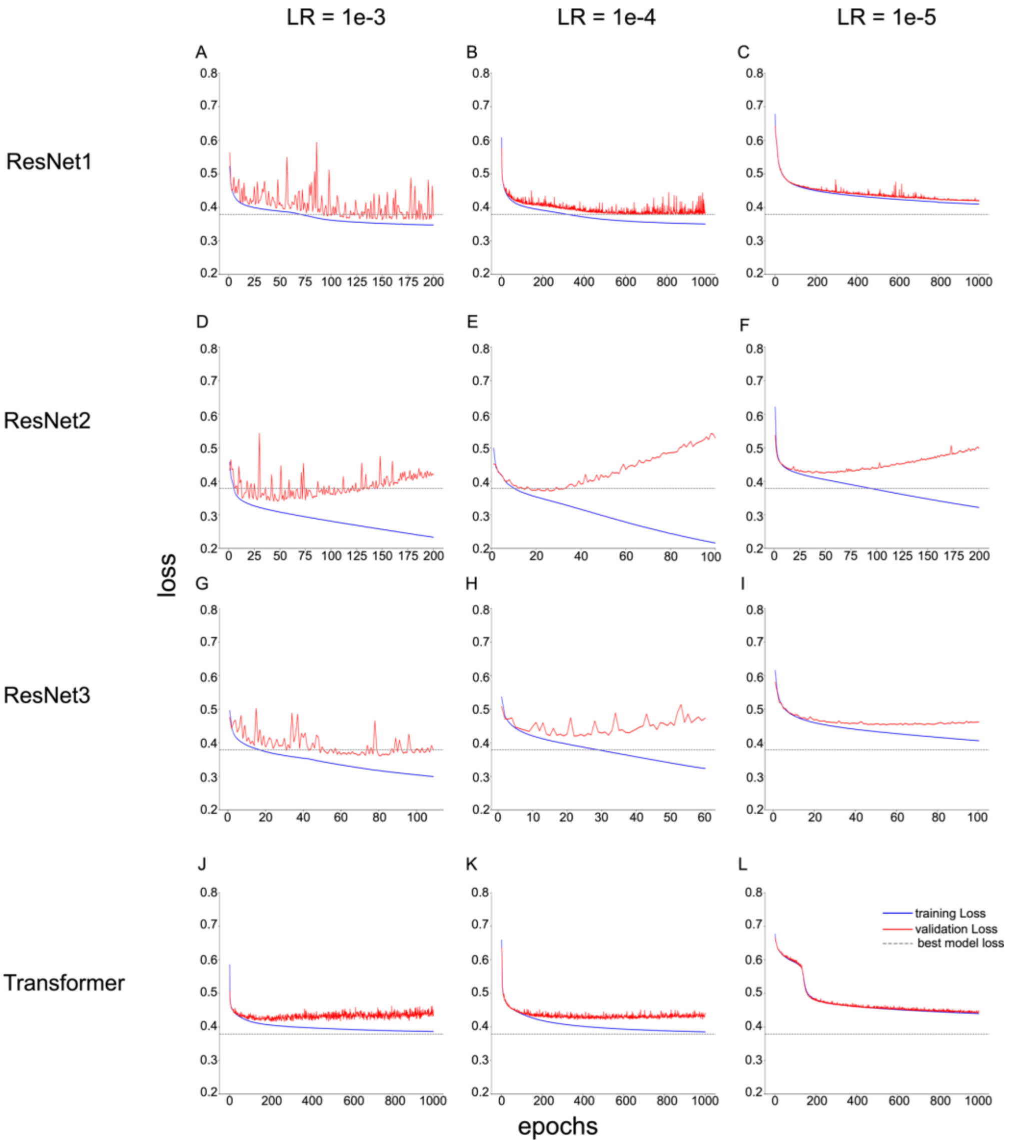

Fig S1. Training and validation loss across deep neural network architectures tested for nanopore squiggle signal-based viability inference

(A-C) Model loss of ResNet1 at learning rates (LRs) of 1e-3, 1e-4, and 1e-5; (D-F) model loss of ResNet2 at LRs of 1e-3, 1e-4, and 1e-5; (G-I) model loss of ResNet3 at LRs of 1e-3, 1e-4, and 1e-5; and (J-L) model loss of the transformer models at LRs of 1e-3, 1e-4, and 1e-5. The solid blue line indicates the training loss, the solid red line indicates the validation loss, and the dashed line indicates the minimum validation loss from the final ResNet1, LR=1e-4, model.

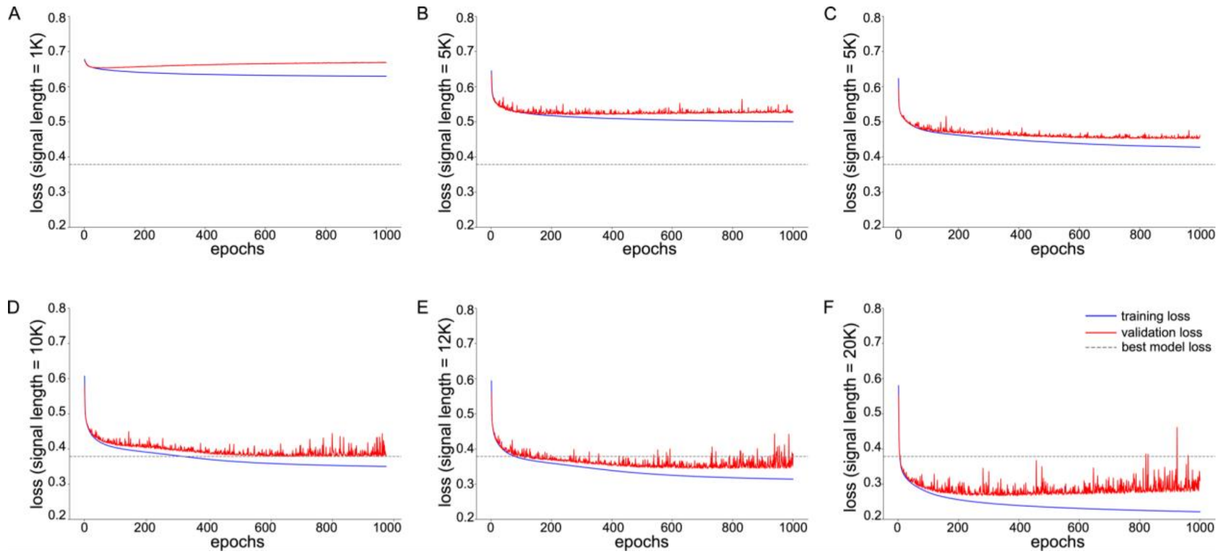

Fig S2. Training and validation loss of ResNet1 at various signal chunk sizes

The signal chunk size varies from (A) 1k, (B) 5k, (C) 7k, (D) 10k, to (E) 12k and (F) 20k. The solid blue line indicates the training loss, the solid red line indicates the validation loss, and the dashed line indicates the minimum validation loss from the final ResNet1model using a signal chunk size of 10k.

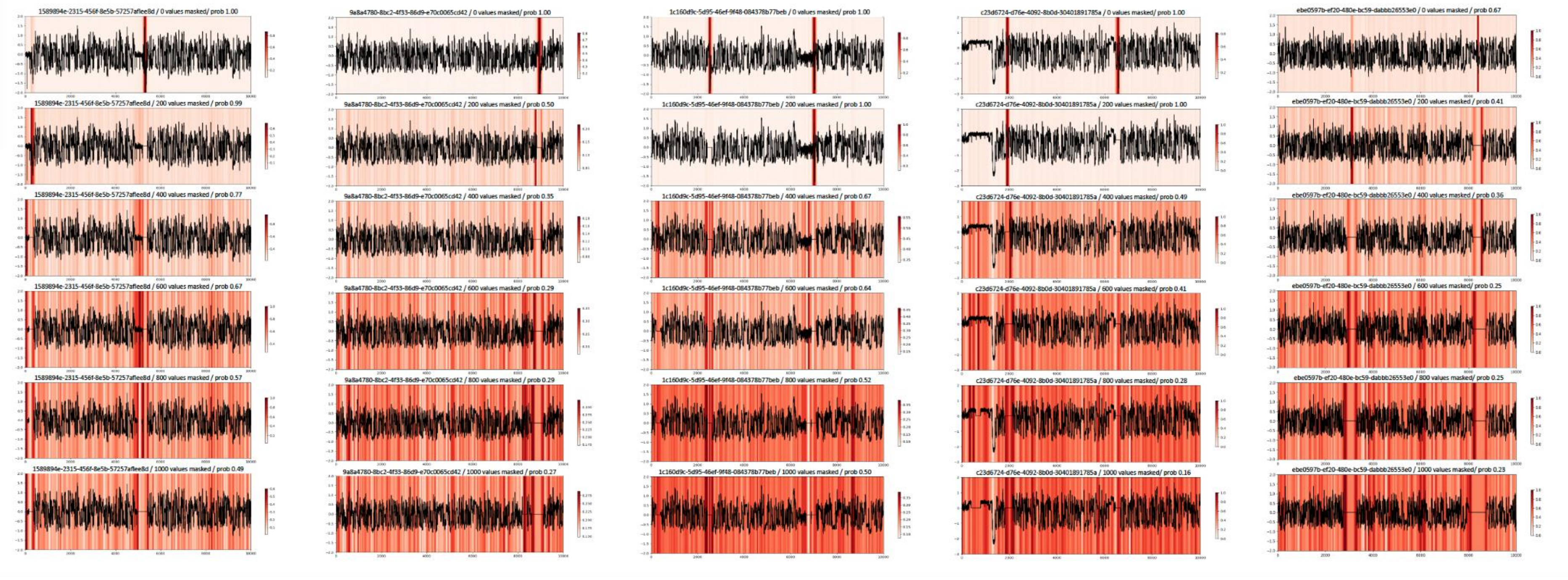

Fig S3. Exemplary drops in ResNet1 prediction probabilities in nanopore signal chunks after consecutive masking of the signal region with the respectively highest CAM value. *Figure headers*: signal chunk ID / total number of masked signal values / prediction probability per signal chunk “prob”. *Left to right*: Five exemplary nanopore signal chunks (length of 10k signals). *Top to bottom*: Consecutive masking of 200 signal values per masking event (Materials and Methods). *Legends*: Red-colored CAM value visualizations; higher CAM values indicate stronger feature map activations.

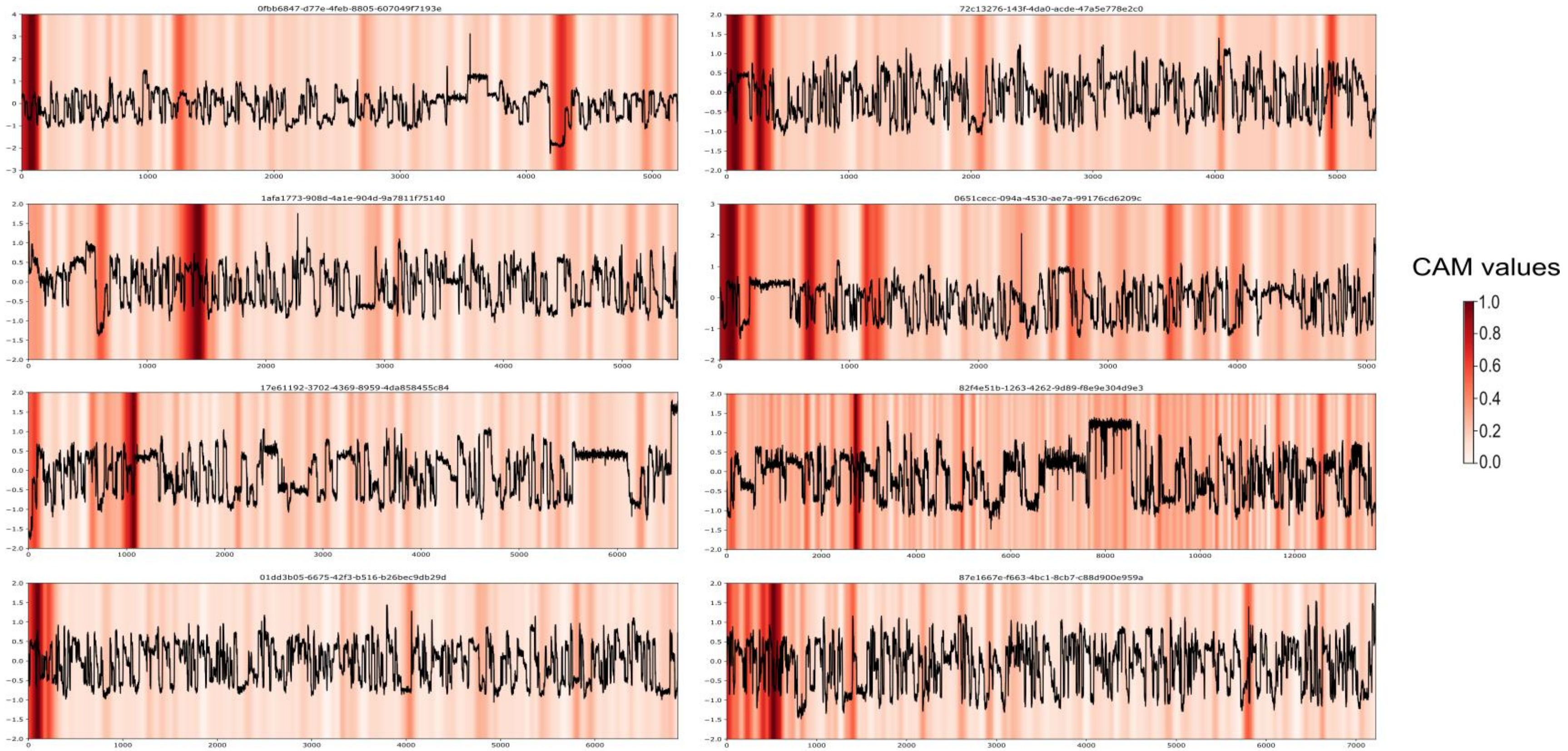

Fig S4. Exemplary nanopore signal patterns of antibiotic-killed *E. coli* sequencing reads and XAI Class Activation Mapping (CAM). *Legend*: Red-colored CAM value visualizations; higher CAM values indicate stronger feature map activations.
