# Supplementary material for: Nanopore- and AI-empowered microbial viability inference
Source: Gigascience. 2025 Sep 3;14:giaf100. doi: 10.1093/gigascience/giaf100 (PMC12405693; doi:10.1093/gigascience/giaf100)

GigaScience

Nanopore- and AI-empowered metagenomic viability inference

--Manuscript Draft--

**Manuscript Number:**	GIGA-D-24-00390
**Full Title:**	Nanopore- and AI-empowered metagenomic viability inference
**Article Type:**	Research
**Funding Information:**	Helmholtz Principal Investigator Grant — Not applicable<br>Munich School for Data Science — Ms Harika Muazzez Ürel<br>BBSRC — Mr. Edward J. Martin<br>STFC Food Network+ Scoping Grant — Mr. Edward J. Martin<br>Helmholtz Association Initiative and Networking Fund — Dr. Sabrina Benassou, Dr. Stefan Kesselheim
**Abstract:**	The ability to differentiate between viable and dead microorganisms in metagenomic samples is crucial for various microbial inferences, ranging from assessing ecosystem functions of environmental microbiomes to inferring the virulence of potential pathogens. While established viability-resolved metagenomic approaches are labor-intensive as well as biased and lacking in sensitivity, we here introduce a new fully computational framework that leverages nanopore sequencing technology to assess microbial viability directly from freely available nanopore signal data. Our approach utilizes deep neural networks to learn features from such raw nanopore signal data that can distinguish DNA from viable and dead microorganisms in a controlled experimental setting. The application of explainable AI tools then allows us to robustly pinpoint the signal patterns in the nanopore raw data that allow the model to make viability predictions at high accuracy. Using the model predictions as well as efficient explainable AI-based rules, we show that our framework can be leveraged in a real-world application to estimate the viability of pathogenic Chlamydia, where traditional culture-based methods suffer from inherently high false negative rates. This application shows that our viability model captures predictive patterns in the nanopore signal that can in principle be utilized to predict viability across taxonomic boundaries and indendent of the killing method used to induce bacterial cell death. While the generalizability of our computational framework needs to be assessed in more detail, we here demonstrate for the first time the potential of analyzing freely available nanopore signal data to infer the viability of microorganisms, with many applications in environmental, veterinary, and clinical settings.
**Corresponding Author:**	Lara Urban<br>Helmholtz Center Munich German Research Center for Environmental Health: Helmholtz Zentrum Munchen Deutsches Forschungszentrum fur Gesundheit und Umwelt<br>Neuherberg, GERMANY
**Corresponding Author Secondary Information:**	
**Corresponding Author's Institution:**	Helmholtz Center Munich German Research Center for Environmental Health: Helmholtz Zentrum Munchen Deutsches Forschungszentrum fur Gesundheit und Umwelt
**Corresponding Author's Secondary Institution:**	
**First Author:**	Harika Muazzez Ürel
**First Author Secondary Information:**	
**Order of Authors:**	Harika Muazzez Ürel<br>Sabrina Benassou<br>Tim Reska<br>Hanna Marti

Powered by Editorial Manager® and ProduXion Manager® from Aries Systems Corporation

	Enrique Rayo
	Edward J. Martin
	Michael Schloter
	James M. Ferguson
	Stefan Kesselheim
	Nicole Borel
	Lara Urban
**Order of Authors Secondary Information:**	
**Additional Information:**	
**Question**	**Response**
Are you submitting this manuscript to a special series or article collection?	No
**Experimental design and statistics**<br><br>Full details of the experimental design and statistical methods used should be given in the Methods section, as detailed in our Minimum Standards Reporting Checklist. Information essential to interpreting the data presented should be made available in the figure legends.<br><br>Have you included all the information requested in your manuscript?	Yes
**Resources**<br><br>A description of all resources used, including antibodies, cell lines, animals and software tools, with enough information to allow them to be uniquely identified, should be included in the Methods section. Authors are strongly encouraged to cite Research Resource Identifiers (RRIDs) for antibodies, model organisms and tools, where possible.<br><br>Have you included the information requested as detailed in our Minimum Standards Reporting Checklist?	Yes
**Availability of data and materials**	Yes

Powered by Editorial Manager® and ProduXion Manager® from Aries Systems Corporation

All datasets and code on which the conclusions of the paper rely must be either included in your submission or deposited in publicly available repositories (where available and ethically appropriate), referencing such data using a unique identifier in the references and in the "Availability of Data and Materials" section of your manuscript.<br><br>Have you have met the above requirement as detailed in our Minimum Standards Reporting Checklist?	

Powered by Editorial Manager® and ProduXion Manager® from Aries Systems Corporation

Click here to access/download;Manuscript;manuscript_GigaScience_Bio.do

Nanopore- and AI-empowered metagenomic viability inference

Harika Urel[1,2,3], Sabrina Benassou[4], Tim Reska[1,2,3], Hanna Marti[5], Enrique Rayo[5], Edward J. Martin[6], Michael Schloter[3,7], James M. Ferguson[8], Stefan Kesselheim[4], Nicole Borel[5], Lara Urban[1,2,3]*

[1] Helmholtz AI, Helmholtz Zentrum Muenchen, Neuherberg, Germany
[2] Helmholtz Pioneer Campus, Helmholtz Zentrum Muenchen, Neuherberg, Germany
[3] Technical University of Munich, School of Life Sciences, Freising, Germany
[4] Jülich Supercomputing Centre, Forschungszentrum Jülich, Germany
[5] Institute of Veterinary Pathology, University of Zürich, Switzerland
[6] Institute of Ecology and Evolution, School of Biological Sciences, University of Edinburgh, United Kingdom
[7] Comparative Microbiome Analysis, Helmholtz Zentrum Muenchen, Neuherberg, Germany
[8] Garvan Institute of Medical Research, Sydney, Australia

*Corresponding author: lara.h.urban@gmail.com

Abstract

The ability to differentiate between viable and dead microorganisms in metagenomic samples is crucial for various microbial inferences, ranging from assessing ecosystem functions of environmental microbiomes to inferring the virulence of potential pathogens. While established viability-resolved metagenomic approaches are labor-intensive as well as biased and lacking in sensitivity, we here introduce a new fully computational framework that leverages nanopore sequencing technology to assess microbial viability directly from freely available nanopore signal data. Our approach utilizes deep neural networks to learn features from such raw nanopore signal data that can distinguish DNA from viable and dead microorganisms in a controlled experimental setting. The application of explainable AI tools then allows us to robustly pinpoint the signal patterns in the nanopore raw data that allow the model to make viability predictions at high accuracy. Using the model predictions as well as efficient explainable AI-based rules, we show that our framework can be leveraged in a real-world application to estimate the viability of pathogenic *Chlamydia*, where traditional culture-based methods suffer from inherently high false negative rates. This application shows that our viability model captures predictive patterns in the nanopore signal that can in principle be utilized to predict viability across taxonomic boundaries and indendent of the killing method used to induce bacterial cell death. While the generalizability of our computational framework needs to be assessed in more detail, we here demonstrate for the first time the potential of analyzing freely available nanopore signal data to infer the viability of microorganisms, with many applications in environmental, veterinary, and clinical settings.

Author summary

Metagenomics investigates the entirety of DNA isolated from an environment or a sample to holistically understand microbial diversity in terms of known and newly discovered microorganisms and their ecosystem functions. Unlike traditional culturing of microorganisms, metagenomics is not able to differentiate between viable and dead microorganisms since DNA

might readily persist under different environmental circumstances. The viability of microorganisms is, however, of importance when making inferences about a microorganism's metabolic potential, a pathogen's virulence, or an entire microbiome's impact on its environment. As existing viability-resolved metagenomic approaches are labor-intensive, expensive, and lack sensitivity, we here investigate our hypothesis if freely available nanopore sequencing signal data, which captures DNA molecule information beyond the DNA sequence, might be leveraged to infer such viability. This hypothesis assumes that DNA from dead microorganisms accumulates certain damage signatures that reflect microbial viability and can be read from nanopore signal data using fully computational frameworks. We here show first evidence that such a computational framework might be feasible by training a deep model on controlled experimental data to predict viability at high accuracy, exploring what the model has learned, and applying it to an independent real-world dataset of an infectious pathogen. While the generalizability of this computational framework needs to be assessed in much more detail, we demonstrate that freely available data might be usable for relevant viability inferences in environmental, veterinary, and clinical settings.

Introduction

While microbial cultivation remains a foundational technique in microbiology to assess the taxonomic composition of microbial communities and to properly understand their physiology and ecosystem functions [1], only a small fraction of microbial diversity has been isolated in pure culture [2]. This limitation has led to undiscovered functions and biased representations of the phylogenetic diversity of microbial communities in nearly all of Earth's environments [2]. While medically relevant microorganisms of the human microbiome constitute an exemption since they have been disproportionately well studied through microbial cultures [3], the clinical application of microbial cultivation for pathogen profiling is also limited due to its time-consuming and labor-intensive nature [4].

The first studies of the so-called "microbial dark matter" have been enabled by advances in culture-independent molecular methodology [5], and have been based on amplifications of conserved marker regions such as ribosomal RNA genes [6]. Such targeted metabarcoding approaches, however, suffer from several limitations: They can often not provide strain- or even species-level taxonomic resolution, are highly dependent on genomic database completeness, do not allow for any functional inferences or virulence annotations, and often introduce amplification bias due to differential amplification efficiency and primer mismatches, which can significantly distort the representation of microbial community compositions [7].

Metagenomics, on the other hand, is a shotgun sequencing-based molecular methodology that can assess the entirety of DNA isolated from an environment or a sample and *de novo* assemblies of potentially complete microbial genomes of all present microorganisms; such genome-based approaches provide a variety of phylogenetically informative sequences for taxonomic classification, information about the metabolic and virulence potential of microorganisms, and the potential to identify completely novel genes [8, 9].

Especially long-read metagenomic approaches have shown great promise in achieving highly contiguous *de novo* assemblies through the recovery of high-quality metagenome-assembled genomes (MAGs) from complex environments; specifically, the latest advances in nanopore sequencing technologies have resulted in high sequencing accuracies of very long sequencing reads of up to millions of bases, which allowed for the generation of hundreds of MAGs from metagenomic data, including the generation of closed circularized genomes [10, 11].

Nanopore sequencing technology is based on the interpretation of the disruption of an ionic current due to a motor protein guiding individual nucleotide strands through nanopores embedded in an electrically resistant polymer membrane at a consistent translocation speed [12]. This raw nanopore signal, or “squiggle” data, can then be translated into nucleotide sequence using bespoke neural network-based basecalling algorithms [13], which – when efficiently embedded on powerful GPUs – can generate genomic data in real-time. The portable character and straightforward implementation of nanopore sequencing at very upfront investment costs further make this technology accessible for fast microbial and pathogens assessments at point of interest all around the world, including in low- and middle-income countries [14].

In contrast to cultivation-based approaches, molecular methods suffer from their inherent deficiency of not being able to differentiate between viable and dead microorganisms [2, 15]. While cultivation-based approaches only detect viable microorganisms, DNA might remain intact and therefore accessible by molecular methods despite the respective microorganisms being dead [15]. This might be especially relevant in the context of infection prevention and control and pathogen monitoring in the clinical setting, where certain disinfection methods or the use of systemic antibiotics often kill the bacteria before the DNA is destroyed [15, 16], but also for understanding the ecosystem functions of thus far understudied microbiomes [2]: For example, the air microbiome has been shown to be remarkable diverse and variable when assessed through nanopore metagenomics [17], but given the low biomass of this environment it is expected that many microorganisms might be dead and stem from adjacent environments such as soil or water. The persistence of the DNA of dead microorganisms in the environment might hereby depend on many factors, including external conditions such as temperature, pH, and microbial activity, and internal, taxon-specific parameters such as microbial cell wall composition. Viability-resolved metagenomics would, however, be crucial for the interpretation of metagenomic data, ranging from outbreak source detection [18], food safety [19] and public health investigations [20], to ecosystem function inferences [21].

To assess microbial viability from genomic data, several approaches have been developed: Culture-dependent viability methods combine the advantages of cultivation-based and molecular approaches by growing certain microorganisms of interest on selective media; this approach, however, remains time-consuming and labor-intensive and suffers from the same selectivity of growth media and culturable microorganisms as purely cultivation-based approaches [22], especially for fastidious or obligate intracellular microorganisms [23]. Microbial viability can further be assessed through metabolic activity, where microbial cells are incubated with specific substrates whose metabolization produces detectable signals such as ATP production, tetrazolium salt reduction, and radiolabeled substrate incorporation; this approach may, however, not be applicable to all microbial taxa and environmental conditions, and the results might be heavily influenced by the physiological state of the microbial cells [24]. While RNA has been used as a viable/dead marker due to its intrinsic instability [25, 26, 27], the metatranscriptome has to be stable enough to be detectable in certain environments, but unstable enough to reflect viability; if only one gene is targeted, the gene analyzed further has to be continuously expressed [15]. Further issues might arise from the relatively challenging extraction protocols due to the RNA’s instability, and from the relatively evolutionary conservation of gene sequences, which might hamper taxonomic resolution.

Finally, an aspect that can be used for viability-resolved metagenomics is the physical difference between viable and dead cells: Viability PCR (vPCR) uses DNA-intercalating dyes such as ethidium monoazide (EMA) or propidium monoazide (PMA) to differentiate between

viable and dead cells. These dyes penetrate only dead cells with compromised membranes and bind to their DNA via covalent bonds upon photoactivation, preventing it from being amplified during subsequent PCR [15, 16]; this approach has been applied to a diverse array of Gram-negative and -positive bacteria, and to assess the effectiveness of disinfection and heat treatment [24]. It, however, relies on the assumption that membrane integrity is a reliable indicator of viability, which can lead to overestimation of viability if cells lose viability without immediate membrane compromise [28] and can be biased by the dye's variable permeability across different microbial cell wall structures [29, 30]. The dependence of the approach on photoactivation further means that turbid material might hamper the efficiency of the dye [31].

All these established viability-resolved metagenomic approaches are labor-intensive, require additional reagents and sample processing, and are often biased and lack sensitivity. We here hypothesized that the raw, freely available nanopore signal from metagenomic datasets might be leveraged to infer microbial viability, assuming that the native DNA from dead microorganisms accumulates detectable squiggle signatures due to, e.g., external damage, the lack of DNA repair mechanisms, or the enzymatic activity [32, 33, 34]. Such an analysis framework would be fully computational and utilize squiggle data that is automatically obtained with nanopore sequencing. While raw nanopore data is known to contain information about epigenetic modifications [35, 36, 37] and oxidative stress at specific human telomere sites [38], the applicability to assess microbial viability has not yet been tested.

In this study, we produced experimental nanopore sequencing data from viable and dead *Escherichia coli* cultures to optimize deep neural networks to predict viability just from the nanopore squiggle signal. We then applied explainable AI (XAI) tools, which allow us to identify the specific nanopore signal patterns in the input data that allow the model to deliver high-accuracy predictions as an output. We finally showed that our computational framework can be leveraged in a real-world application to estimate the viability of pathogenic *Chlamydia abortus*, an obligate intracellular bacterial species with a complex biphasic lifecycle causing enzootic abortion in sheep and goats [39]. Together, we show that our framework can in principle predict viability across taxonomic boundaries and independent of the killing method used to induce bacterial cell death. While we have not yet tested the generalizability of our computational framework, we here demonstrate for the first time the potential of analyzing freely available nanopore signal data to infer the viability of microorganisms, with many applications in environmental, veterinary, and clinical settings.

Results & Discussion

Neural Network Training

We generated controlled training data by nanopore sequencing native DNA of viable and dead *E. coli* (Materials and Methods). We killed *E. coli* cultures using different stressors to then isolate the extracellular DNA and expose it to natural degradation. We only obtained enough DNA for subsequent shotgun sequencing from the viable culture and from the culture killed through rapid UV exposure (viable: 212 ng/µL; UV: 5.46 ng/µL; heat shock: 0.03 ng/µL; bead beating: 0.67 ng/µL; Materials and Methods). We repeated this experiment and confirmed that rapid heat shock as well as bead beating exposure again resulted in very low DNA concentrations, suggesting quick and complete DNA degradation. We hypothesize that UV exposure constitutes the only stressor that simultaneously destroys bacterial cell walls as well as inactivates DNA-degrading enzymes. In the case of killing by heat shock and bead beating, such enzymes might have remained active and would have been carried forward by our

extracellular DNA isolation approaches (Materials and Methods) to then degrade all genomic material during natural exposure. We therefore created nanopore shotgun sequencing of the viable and the UV-exposed culture, which resulted in 2.92 Gbases (Gb; median read length of 2,476 b) and 2.69 Gb (median read length of 1,606 b) of sequencing output, respectively (Materials and Methods).

We then tested the implementation of different neural network architectures to predict the binary viability state from the raw nanopore data (0=viable; 1=dead; Materials and Methods). We processed the *E. coli* nanopore signal, or "squiggle", data, cut it into altogether 3,181,600 signal chunks of 10k signals, and separated the chunks into balanced training (60%), validation (20%), and test (20%) set along each original sequencing read to avoid that signal chunks from the same read would end up in the same dataset (Materials and Methods). These signal chunks can be treated as 1D time series signal data of consistent length. We trained the different model architectures using different learning rates (LRs) up to 1,000 epochs, assessing the models' performance based on training and validation loss after each epoch (Materials and Methods; **Table S1**; **Fig S1**). The loss plot of our best-performing model, a residual neural network with convolutional input layers (configuration ResNet1; LR=1e-4; **Table S1**; **Fig S1**; Materials and Methods) shows minimal overfitting when the minimum validation loss is reached at epoch 667 (**Fig 1A**). The other residual neural network architectures (ResNet2, ResNet3), on the other hand, resulted in overfitting to the training data at any LR, and the transformer architecture did not reach the minimum validation loss of ResNet1 (**Fig S1**). We next only focused on ResNet1 and optimized its probability threshold using the validation set; in order to obtain a high accuracy and F1 score, we maintained the probability threshold at the default value of 0.5 (**Fig 1B**), which resulted in a good final performance on the test data with an accuracy of 0.83 and a F1 score of 0.81 (**Fig 1B**, *inlet*) as well as Area Under the Curve (AUC) values of 0.90 (Area Under the Receiver Operating Characteristic curve; AUROC) and 0.92 (Area Under the Precision-Recall curve; AUPR), respectively (**Fig 1C**).

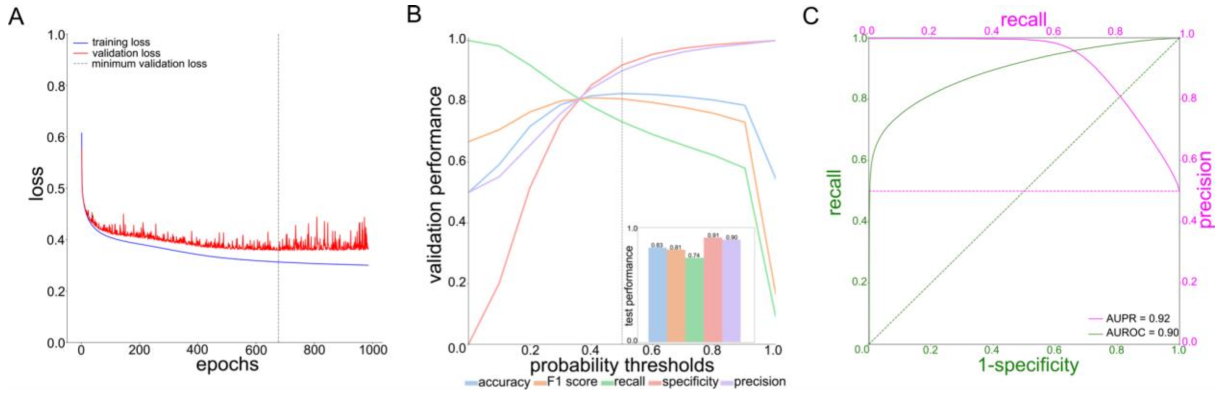

Fig 1. Training of the Viability Residual Neural Network (ResNet)

(A) Model loss for validation and test sets across 1,000 epochs; the minimum validation loss of the ResNet was reached at epoch 677. (B) Prediction probability threshold optimization on the validation dataset resulted in a probability threshold of 0.5 for obtaining maximum accuracy and F1 score. *Inlet:* Performance of the final ResNet model ResNet1 on the test data (Materials and Methods). (C) Test performance of the ResNet1 in terms of Precision-Recall (PR; *pink)* and Receiver Operating Characteristic (ROC; *green*) curves and their respective Areas Under the Curve (AUPR, AUROC).

We also trained the same residual neural network architecture ResNet1 on the basecalled nanopore data of viable and dead *E. coli* at a standardized chunk size of 800 b, which roughly

corresponds to a signal chunk size of 10k signals (Materials and Methods). Independently of if we only basecalled the canonical bases or used a N6-methyladenine (6mA) modification-aware basecalling model (Materials and Methods), the model could not be trained to distinguish viable from dead data just from DNA sequence data (**Table S1**). This shows that our model captures patterns in the squiggle data that goes beyond the encoding of nucleotides and their known epigenetic modifications. While this was expected since we used the same *E. coli* culture with the same reference genome to create the viable and dead datasets, we hereby ruled out that our squiggle-based model captured any random differences in DNA sequence context between the two datasets that might have occurred by chance.

We additionally obtained the performance of ResNet1 for different signal chunk sizes (**Fig S2**; **Table S1**), and we found that viability prediction performance was possible from a minimum chunk size of approximately 5k, but can be further improved with increasing chunk size. This shows that larger signal chunks contain more information that can be used by our model to make accurate per-chunk predictions while the resulting reduced size of the dataset, especially of the training dataset, did not influence the learning ability of the model. While this might be valuable information for future applications where potentially the full length of a read could be leveraged, we here decided to stick to a signal chunk size of 10k signals, which had already resulted in good performance (**Fig 1**; **Table S1**) and which can be applied to relatively small sequencing reads as is often the case for metagenomic datasets (e.g., [17]).

Explainable AI

We implemented Class Activation Maps (CAM) as an XAI method [40] to identify the most important regions in the nanopore signal data that inform the model's viability classifications (Materials and Methods; **Fig 2A**). We found that "dead" signal chunks exhibited discrete regions of increased CAM values ("CAM regions" defined at CAM values>0.8; **Fig 2B** for several true positive classifications of the test dataset). To confirm the importance of these CAM regions for the model's final predictions, we applied consecutive masking of the regions with the highest CAM values within each nanopore signal chunk (**Fig S3** for several examples); we observed that the prediction probability for being classified as "dead" decreased with increased masking of CAM-relevant regions, either by consecutively masking regions using a consistent mask size or by increasing the mask size (from 100 to 2k signals; **Fig 2C**). This shows that the CAM application reliably pinpoints patterns in the nanopore signal that are predictive for our viability model.

We used the CAM regions to manually investigate the squiggle signals, and found that many CAM regions of "dead" signal chunks included a sudden substantial drop in the nanopore signal. We therefore developed a simple algorithm that identifies such sudden drops, and applied this XAI rule to our test dataset (Materials and Methods; **Fig 2D**). We here classified any signal chunk with at least one sudden drop as "dead", and all others as "viable". While this simplified algorithm led to a drop in overall performance, we could still reach a relatively good overall accuracy of 0.68 (in comparison to 0.83 of the full model; **Fig 2E**). While the XAI rule maintained performance of specificity and precision, we observed a substantial drop in recall in comparison to the full model (now 0.39 instead of 0.74). This shows that while the absence of a sudden drop in the nanopore signal data seems to reliably predict viability, not all "dead" signal chunks seem to contain such a sudden drop. While this sudden-drop detection still seems to be at the core of our model's interpretability (when focusing on high-confidence true positive chunks at $p>0.99$, the recall increased to 0.68), the model seems to additionally detect

more subtle patterns in the nanopore signal data which allow it to increase recall while maintaining specificity and precision.

Based on our previous experience with squiggle data analysis [41, 42], we hypothesize that the substantial sudden drops in nanopore signal might be caused by a twist or kink in the DNA backbone, for example from 6-4pp pyrimidine dimers. The drop would then mark the event of a pore getting blocked due to such damage. Such a twist could also lead to a stalling signal if it impairs the motor protein from processing the DNA strand, which we indeed partially observed in our data (e.g., top signal chunk of **Fig 2B/D**). While more detailed future squiggle analyses as well as application of our model to other viability studies will hopefully shed more light on the biological, chemical, and physical features detected by our model, we conclude that in our current study both, UV exposure (*E. coli*) and heat shock (*Chlamydia*) might have caused such twists in the DNA backbone.

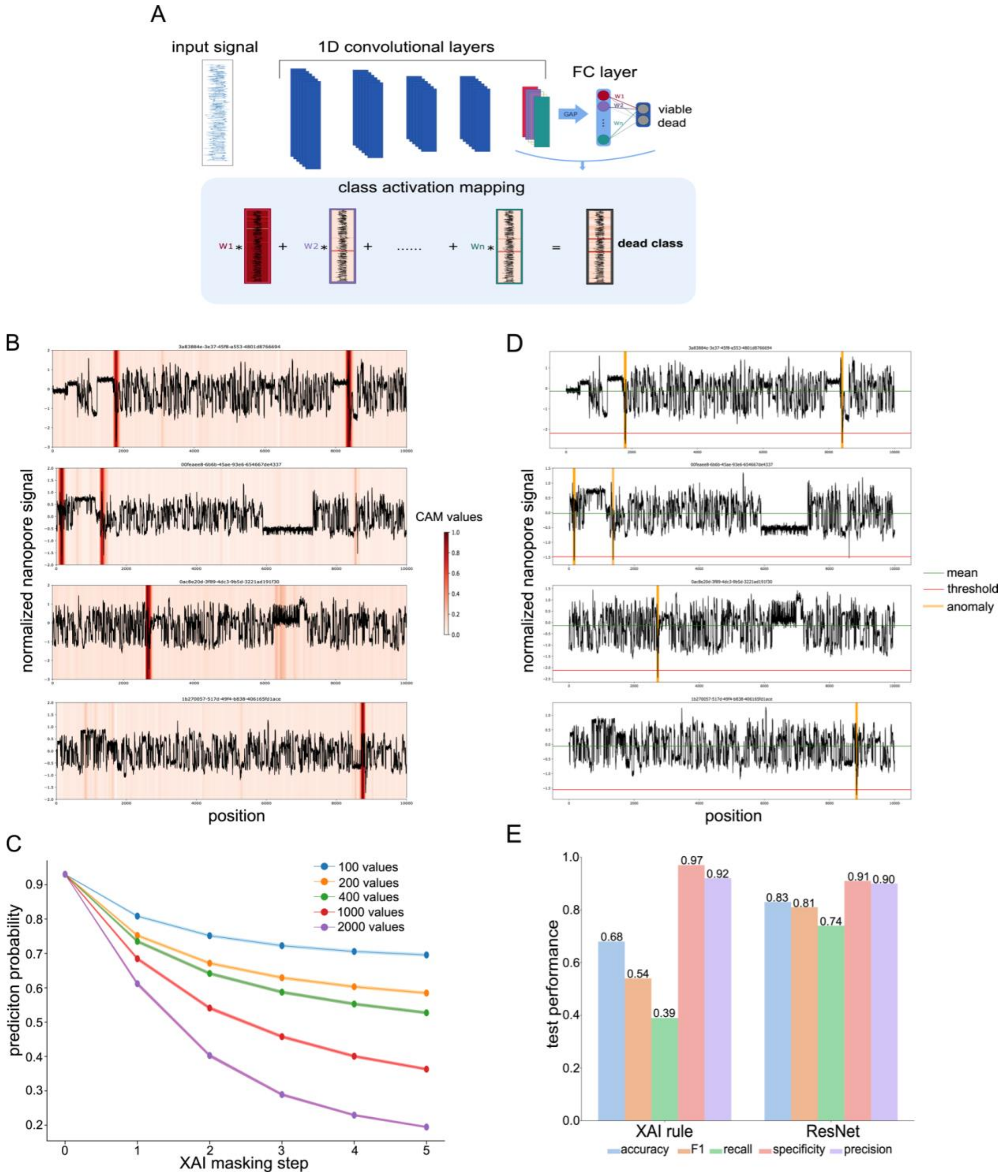

Fig 2. XAI for Interpretability of the Viability ResNet.

(A) The Class Activation Maps (CAMs) leverage the global average pooling (GAP) layer right before the fully connected (FC) layers of the residual neural network to map model interpretability onto the input features; they are generated by aggregating the final convolutional layer's feature maps through a weighted sum, highlighting nanopore signal regions that allow the neural network to make accurate predictions. (B) Exemplary nanopore signal chunks that were classified as "dead" at a prediction probability of $p>0.99$, and their CAM values. Higher CAM values indicate stronger feature map activations. (C) Impact of consecutive masking ($n=5$) of the signal region with the highest CAM value per signal chunk (x-axis) on the model's prediction probability (y-axis); five different mask sizes (from 100 to 2k signals) were used. (D) Application of a simplified XAI rule that classifies signal chunks according to the presence of a "sudden drop" (green: mean signal per chunk; red: threshold for sudden drop definition; yellow: identification of sudden drops in the exemplary signal chunks). (E) Comparison of the performance of the full model (ResNet) with the simplified XAI rule based on the entire test dataset.

Application to pathogenic Chlamydia

We next applied our computational viability framework to distinguish viable from dead *Chlamydia abortus*, one of the most common causes of infectious abortion in small ruminants worldwide [43, 44], and a zoonotic pathogen causing pneumonia and miscarriage in humans [45]. *C. abortus* are obligate intracellular bacterial species with a complex biphasic life cycle that form intracellular vacuoles termed inclusions. These unique properties render both cultivation- and vPCR-based approaches for viability estimations complicated [46]. In order to apply our computational viability framework to this pathogen, we decided to use two differently treated samples of the same strain of *C. abortus* samples for which both cultivation- and vPCR-based approaches had predicted viability with high certainty. We chose a “viable” and a heat-treated (“dead”) sample of the same culture for which we could ascertain viability and non-viability, respectively (Materials and Methods); heat treatment was used since it constitutes the standard approach for killing *C. abortus* [46]. Briefly, for ascertaining viability and non-viability of the two samples, respectively, we applied cultivation and propidium monoazide (PMA)-based vPCR. In the case of cultivation, heat-treated *C. abortus* were unable to form viable inclusions in cell culture, whereas the untreated sample showed high infectivity with 2.6e6 inclusion forming units per mL (IFU/ml). In the case of vPCR, both samples were treated with and without PMA enhancer with or without PMA [46] to then quantify the respective amounts of chlamydial DNA with a sensitive *Chlamydia*-specific qPCR (47; Materials and Methods). The difference in quantity between PMA-treated and untreated DNA was expressed as Δlog10 *Chlamydia* per mL, resulting in 0.42 and 4.2 Δlog10 *Chlamydia* per mL for the control and the heat-treated sample, respectively (**Table 1**). These data are comparable to a previous study in which fresh *C. trachomatis* culture was heat-killed and a viability ratio determined ranging from 0% to 100% resulting in a 3.01 and 0.37 Δlog10 *Chlamydia* per mL for 0% and 100% viable, respectively [46]. Both cultivation and vPCR therefore confirmed that heat-treatment had completely inactivated previously viable *C. abortus* for subsequent culture and had strongly reduced the amount of “viable” DNA using vPCR.

Table 1. Viability PCR (vPCR) results of a “viable” and a “dead” *C. abortus* sample.
A viable and dead (heat-treated at 95°C for 10 min) sample of the same *Chlamydia* (*C. abortus* S26/3) stock were tested for viability using vPCR: PMA-untreated vPCR reflects total *Chlamydia* content; PMA-treated vPCR reflects viable *Chlamydia* content; IFU describes the number of Inclusion Forming Units.

Condition	Titer [IFU/mL]	PMA-untreated vPCR [copy number per mL]	PMA-treated vPCR [copy number per mL]	Viability ratio [%]
viable	$2.67e^{7}$	$4.59e^{7}$	$1.73e^{7}$	37.6
dead	0	$9.03e^{7}$	$5.52e^{7}$	0.01

We next created nanopore shotgun sequencing of these “viable” and “dead” *C. abortus* samples, which resulted in 37.50 Mb (median read length of 1,458 b) and 10.38 Mb (median

read length of 496 b) of sequencing output, respectively (Materials and Methods). We subsequently confirmed that the sequencing data was indeed created from the low-concentration *C. abortus* samples, and not from any contaminating microorganisms: The *de novo* assembly resulted in 18 and 39 contigs, respectively, which all mapped to *C. abortus* (NCBI nt taxonomy id; 83555; Materials and Methods).

We processed this nanopore sequencing data into 42,335 and 13,312 nanopore signal chunks of 10k signal length for the viable and dead sample, respectively. We used our model (ResNet1) to make computational viability predictions on this dataset, which resulted in an accuracy of 0.85 and a F1 score of 0.68 at the previously optimized prediction probability threshold of 0.5. These performance values for the *C. abortus* application are therefore comparable to the performance on the original test *E. coli* data, while the microorganism, killing method, and DNA extractions methods were substantially different (**Fig 3A**; *top row*). While our model's prediction probability distributions across signal chunks (**Fig 3A**; *bottom row*) were comparable between viable *E. coli* and *C. abortus*, dead *C. abortus* resulted in a more bimodal distribution than dead *E. coli*, with several "dead" chunks receiving close-to-zero prediction probabilities, suggesting viability (**Fig 3A**; *bottom row*; *right column*). The probability threshold-independent application of our model to *C. abortus* consequently resulted in decreased AUC values (AUROC of 0.80 and AUPR of 0.64; **Fig 3B**) in comparison to its application to *E. coli* (AUROC of 0.90 and AUPR of 0.92; **Fig 1C**). While this difference in performance might just mean that ResNet1 is less sensitive at detecting "dead" signal chunks in the *C. abortus* data than in the *E. coli* data that it was trained on, it might also reflect true variability: The *C. abortus* sample that was nanopore-sequenced to create "dead" nanopore signal data had strongly reduced amounts of "viable" DNA according to the vPCR results but might have still contained DNA from viable *C. abortus* bacterial cells – different from our dead *E. coli* sample which was stringently filtered to only contain extracellular DNA and subsequently subjected to natural degradation (Materials and Methods). The latter hypothesis might be supported by the observation that the application of our CAM-based XAI to the "dead" *C. abortus* signal chunks detected similar patterns as in the "dead" *E. coli* signal chunks (**Fig 3C**). An application of our XAI rule (Materials and Methods) further resulted in similar performance as its application to the *E. coli* signal chunks with good accuracy (0.78), high specificity (0.91), and low recall (0.36).

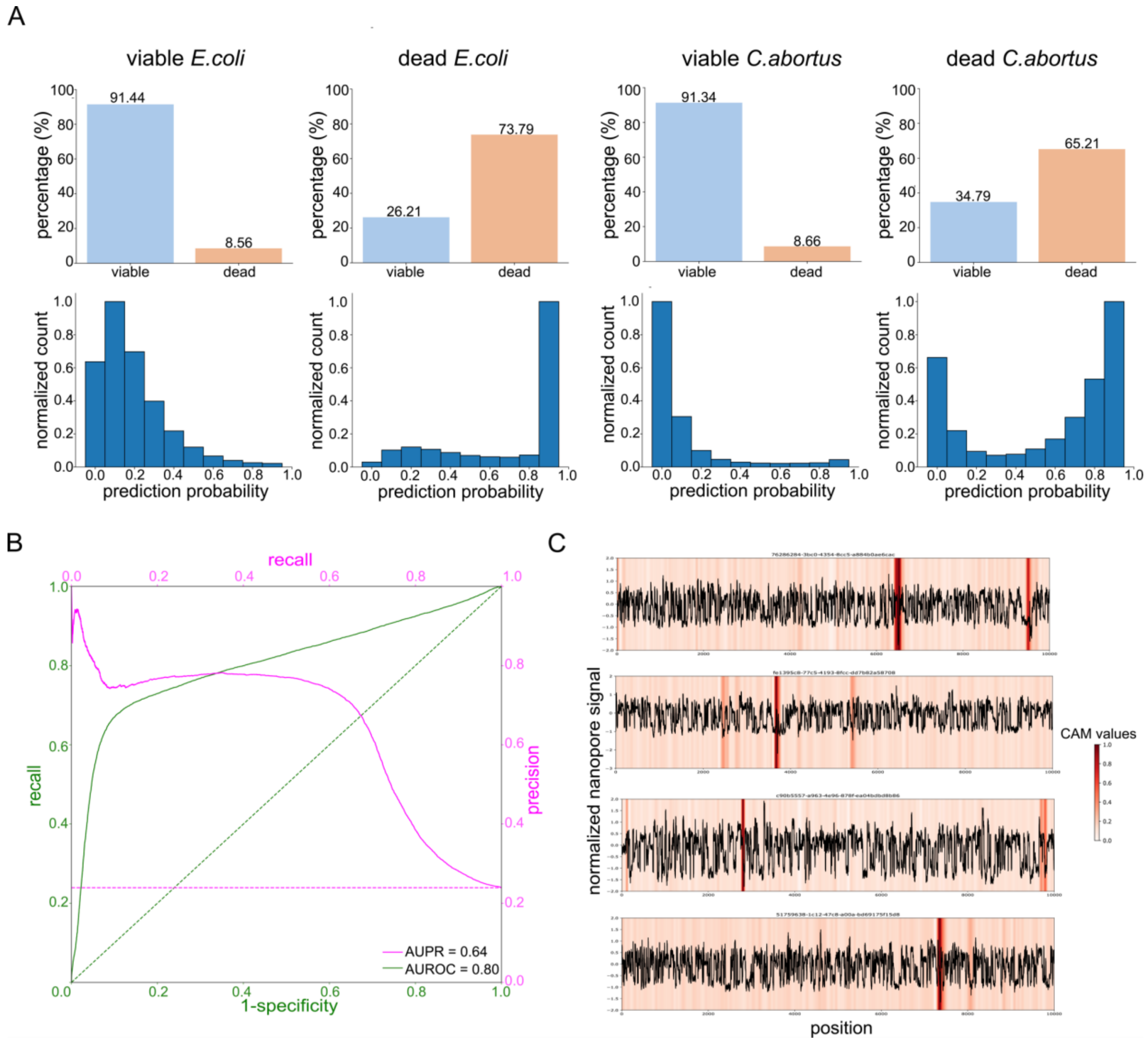

Fig 3. Application of the Viability ResNet to the infectious pathogen *Chlamydia*.
(A) Model performance comparisons between *E. coli* (*left*) and *C. abortus* (*right*) datasets at the optimized prediction probability threshold of 0.5. *Top row*: Binary model predictions for viable and dead *E. coli* and *C. abortus*, respectively; *bottom row*: Normalized distribution of model prediction probabilities across all signal chunks, respectively. For *E. coli*, all test signal chunks are visualized (388k "viable" and "dead" chunks, respectively), and for *C. abortus*, all signal chunks are visualized (42,335 "viable" and 13,312 "dead" chunks). (B) Performance of the model on the *C. abortus* dataset in terms of Precision-Recall (PR; pink) and Receiver Operating Characteristic (ROC; green) curves and their respective Areas Under the Curve (AUPR, AUROC). (C) Exemplary *Chlamydia* nanopore signal chunks that were correctly classified as "dead", and their CAM values. Higher CAM values indicate stronger feature map activations.

AI- and nanopore-empowered viability-resolved metagenomics

While metagenomic approaches provide the unique opportunity of generating *de novo* assemblies and potentially complete microbial genomes to explore the "microbial dark matter" as well as to infer potential functions such as metabolic and virulence potential [8, 9], they have suffered from their inability to differentiate between viable and dead microorganism [2, 15]. Such viability inferences can, however, distort any microbial inference, ranging from assessing ecosystem functions of environmental microbiomes to inferring the virulence of potential pathogens. As established viability-resolved metagenomic approaches are labor-intensive as well as biased and lack sensitivity (e.g., 22), we here show first evidence that a

fully computational framework based on residual neural networks with convolutional data processing layers can leverage raw nanopore signal data, also known as squiggle data, to make accurate inferences about microbial viability (test set accuracy of 0.83).

Our subsequent XAI analyses point to the potential role of DNA backbone damage for achieving accurate model predictions; however, more work is needed to fully understand the biological, physical, or chemical underpinnings of our viability model predictions. First, the simplified XAI rule is not sufficient to correctly classify the majority of “dead” signal chunks (recall of 0.39), which means that the residual neural network has apparently picked up on additional, more ambiguous signal patterns that allow the full model to make more sensitive predictions (recall of 0.74). Besides exploring the underlying rules of such additional signal patterns, we will also apply the viability model to more datasets to assess its generalizability. Especially the probing of different taxonomic groups and killing methods should help us tease apart the origins of our current XAI results. Our study, however, already provides first evidence that our viability model captures predictive patterns in the nanopore signal that can in principle be utilized to predict viability across taxonomic boundaries and independent of the killing method. The application to estimate the viability of pathogenic *Chlamydia* (prediction accuracy of 0.85) is hereby of potentially immediate interest to veterinary scientists since traditional methods for assessing the pathogen’s viability have been labor-intensive and suffered from inherently high false negative rates.

While the generalizability of our model needs to be assessed in much more detail, including for other microbial taxa such as spore-forming bacteria or fungi and for real-world metagenomics applications, the potential benefits of such a widely applicable computational framework could be immense, with many potential applications in environmental, veterinary, and clinical settings. As is the case for epigenetic inferences [35, 36, 37], the viability inference-enabling squiggle data is a complementary output of any nanopore sequencing experiment of native DNA, that is then usually basecalled and archived for future re-basecalling after potential basecalling model improvements. This means that any future nanopore-based metagenomic study could make viability predictions for free without additional costs and laboratory work, and that any existing archived nanopore data could be assessed in terms of its microorganisms’ viability – which would allow us to quantify the impact of dead microorganisms on metagenomics in general, and to further explore factors such as species- and environment-specificity. We finally anticipate that quantitative AI modeling has the potential to inform more differentiated viability assessments, which might help quantify or even time degradation events and decipher the impact of dormancy on metagenomic studies [48, 49].

Materials and Methods

Training Data Generation

We cultured *E. coli K12* in 200 mL Luria-Bertani (LB) medium for 24 hours at 37°C to reach the log phase of the growth curve. The culture was then used to inoculate four 200 mL LB media in 1L Erlenmeyer flasks, which were again incubated for 24 hours to reach the growth log phase. One of the media was used as viable control, i.e. DNA was extracted from 750 uL of the living culture using the spin-column based QIAGEN PowerSoil Pro Kit (QIAGEN, 2018, Hilden, Germany), following the manufacturers' instructions. The remaining three cultures were killed by one of the following stressors: UV irradiation at 254 nm for 15 min, heat shock at 120°C for 5 min, or bead beating for 30 min. To then separate extracellular DNA from cell debris and intact bacterial cells, we centrifuged the media for 10 min at 4,000 x g and filtered the supernatant through 0.2 µm filters. The resulting extracellular DNA was subsequently kept at room temperature for 5 days to simulate the natural accumulation of DNA degradation. DNA from dead bacteria was extracted from these samples using the same extraction approach following the QIAGEN PowerSoil Pro Kit protocol, but the first lysis buffer step was omitted since cell lysis had already happened.

We then used Oxford Nanopore Technologies' Rapid Barcoding library preparation kit (RBK114-24 V14), R10.4.1 MinION flow cells, and MinKNOW software v23.04.5 for shotgun nanopore sequencing of the "viable" and "dead" DNA. We used four barcodes for each sample, resulting in DNA input of 800 ng and 218 ng for the preparation of the "viable" and "dead" library, respectively. We ran each library for 24 h, using two different flow cells to avoid any cross contamination, and filtered the resulting nanopore data at a minimum read length of 20 b. Raw nanopore data was created using the standard translocation speed of 400 b/s, and a sampling frequency of 5 kHz.

We applied Dorado v4.2.0 (https://github.com/nanoporetech/dorado) SUP-basecalling (dna_r10.4.1_e8.2_400bps_sup@v4.2.0) and 6mA-aware SUP-basecalling (6mA@v1) to all nanopore reads that had passed internal data quality thresholds to obtain *E. coli* DNA sequence data. We subsequently removed sequencing adapters and barcodes using Porechop v0.2.3 (https://github.com/rrwick/Porechop).

Neural Network Architecture and Training

We tested the implementation of different residual neural networks and transformer architectures to predict the binary viability state from the raw nanopore data (0=viable; 1=dead). The first residual neural network, ResNet1, consists of four layers, each containing two bottleneck blocks. Each bottleneck block consists of convolutional layers, batch normalization, and a rectified linear unit (ReLu) activation function. Each of the four layers consists of an increasing number of convolutional channels: 20, 30, 45, and 65, respectively, followed by global average pooling and a fully connected layer, resulting in 66,916 parameters. The model then uses a softmax function to convert logits, the raw outputs from the fully connected layer, into predicted probabilities ranging from 0 to 1. We evaluated the training of the model using the Adam optimizer for mini-batch gradient descent at three different LRs (1e-3, 1e-4, and 1e-5), training the model up to 1,000 epochs and at a batch size of 1,000 signal chunks. We initialized the model using Kaiming initialization. For ResNet2, we increased the number of convolutional channels to 40, 60, 90, and 135, respectively, resulting in 1,828,777 parameters. For ResNet3, we increased the number of convolutional channels to 512, 30, 45, and 67, respectively, resulting in 2,479,140 parameters. The transformer model was based on

a positional encoding, convolutional layer with a channel number of 24 and one block of one attention head, resulting 219,586 parameters.

We processed the *E. coli* squiggle data by excluding the first 1,500 signal points (potential noise, adapter sequences, or barcodes), then cutting it into signal chunks of 10k signals, and separated the chunks into balanced training (60%), validation (20%), and test (20%) set along each original sequencing read to avoid that signal chunks from the same read would end up in the same dataset. We pooled the viable and dead signals chunks to obtain exactly balanced training, validation, and test sets. For normalizing each chunk, we subtracted the median per chunk and divided it by the median absolute deviation (MAD) to make the signal data robust to outliers. We then scaled the signal by the MAD scaling factor 1.4826, and replaced outliers exceeding 3.5 times the scaled MAD by the mean of their two neighboring values.

We also trained ResNet1 on the basecalled nanopore data (with or without 6mA basecalling) of viable and dead *E. coli* at a standardized chunk size of 800 b, which roughly corresponds to a signal chunk size of 10k signals. For encoding, we used a one-hot encoding method to turn DNA sequence into unique binary vectors. We then concatenated and saved these encoded sequences as tensors for training and testing. We finally trained ResNet1 on signal chunks of different signal lengths, ranging from 1k to 20k signals.

Explainable AI

We utilized CAMs to identify and visualize signals regions that influenced the model's decision-making. As feature maps from the final convolutional layer undergo a global average pooling layer where each map is averaged and concatenated, we can calculate the weighted sum of these feature maps using the weights of the fully connected layer and project it back onto the preprocessed signal [40]. To do so, we implemented CAM in Python/PyTorch. During the forward pass, we ensure that the feature maps from the last convolutional layer are captured. To compute the CAM, we use the weights of the model's output layer for the class of interest, multiplying these weights with the corresponding feature maps and then summing them up. We convert the resulting CAM to an array and normalize its values to a range of 0 to 1. We then overlay the CAM on the original input signal to identify the regions most influential in the model's decision-making process. We additionally used the Remora API to match raw nanopore data to the corresponding Dorado-basecalled bases, to then manually investigate any obvious sequence abnormalities in the CAM regions.

For consecutive masking of the CAM regions with the highest CAM values, we used Python to first obtain and normalize the CAM values of all true-positive signal chunks at $p>0.5$ ($n=286{,}179$), identify the maximum value, mask the signal region (i.e., setting to zero after MAD-normlization) at a specified mask size (between 100 and 2k signals) centered around the maximum-CAM signal, and obtain updated prediction probabilities. We repeated this masking step five times and calculated the confidence interval at each masking step: We obtained the mean and Standard Error of the Mean (SEM) of the newly calculated prediction probabilities across all signal chunks to calculate the 95% confidence interval at mean ± 1.96 * SEM. We plotted the results using matplotlib.

We next used Python to develop an algorithm to obtain a simplified XAI rule to distinguish “dead” from “viable” signals chunks based on our CAM results by identifying the presence of at least one sudden drop in the chunk. To identify those sudden drops, we first calculated the mean and standard deviation (SD) of each signal chunk, and found that a scaling factor of 3 identified most manually detected sudden drops at a vertical threshold of mean - 3 * SD.

Application to pathogenic Chlamydia

The *Chlamydia abortus* strain S26/3 associated with ovine abortion (provided by Dr. G.E. Jones, Moredun Research Institute, Edinburgh, UK) was cultured as described by Borel *et al.* [50]. Briefly, the strain was first grown in embryonated chicken eggs and stored at -80°C following 1:2 dilution in sucrose-phosphate-glutamate buffer (SPG). For propagation, the strain was cultured in HEp-2 cells (ATCC CCL-23). Infectious elementary bodies were separated from cell debris as well as non-infectious chlamydial reticulate bodies using a renografin density gradient [51], resuspended in SPG, and stored in aliquots at -80°C. To determine the viability of this stock, one aliquot was thawed on ice and separated into two tubes of which one was heat-treated for 10 min at 95°C. Both the control (“viable”) and the heat-treated (“dead”) samples were then divided into subsamples, which were subsequently used for cultivation, vPCR, and nanopore sequencing.

For viability determination by culture, 100 µl per sample was used to infect two glass coverslips (13 mm in diameter, ThermoScientific, Waltham, MA, USA) in 24-well plates (TPP Techno Plastic Product AG, Trasadingen, Switzerland) seeded to confluence with LLC-MK2 cells (rhesus monkey kidney cell line; provided by IZSLER, Brescia, Italy) [52]. Following inoculation of the monolayer, infection was enhanced by centrifugation for 1 h at 25°C (1000 x g). After 48 h of incubation at 37°C (5% CO_2), cultures were fixed for 10 min in ice-cold methanol. Coverslips were then processed using a well-established immunofluorescence assay [53]. Briefly, DNA was stained with 1 µg/mL 4', 6-diamidino-2’-phenylindole dihydrochloride (DAPI, Molecular Probes, Eugene, OR, USA). In parallel, inclusions were labeled with a *Chlamydiaceae*-specific primary antibody (*Chlamydiaceae* LPS, Clone ACI-P; Progen, Germany), which was diluted 1:200 in blocking solution consisting of 1% bovine serum albumin (BSA, St. Louis, MO, USA) in phosphate-buffered saline (PBS, GIBCO, Invitrogen, Carlsbad, CA, USA). Inclusions were then visualized with Alexa Fluor 488 goat anti-mouse (Molecular Probes) diluted 1:500 in blocking solution. As a final step, coverslips were washed with PBS, mounted with FluoreGuard (Hard Set; ScyTek Laboratories Inc., Logan, UT, USA) on glass slides, and inclusions determined using a Leica DMLB fluorescence microscope (Leica Microsystems, Wetzlar, Germany) and a 10X ocular objective (Leica L-Plan 10x/ 25 M, Leica Microsystems). In parallel, a three-fold dilution series of the sample was performed in 96-well plates (TPP) and processed as above. The number of IFU/ml was then determined using the Nikon Ti Eclipse epifluorescence microscope (Nikon, Tokyo, Japan) at a 20X magnification [52].

For vPCR, two 100 µl subsamples were taken and mixed with 200 µl SPG and 100 µl PMA enhancer for Gram Negative Bacteria (5X, Biotium, Fremont, CA, USA). PMAxx (Biotium) at a final concentration of 50 µM was added (“PMA-treated”) or not (“untreated”) to the subsamples. Samples were then exposed to a 650-W light source using a PMA-Lite LED Photolysis Device (Biotium) for 5 min, followed by 2 min on ice and additional light exposure for 5 min [46].

For vPCR as well as nanopore sequencing, DNA was extracted using the DNeasy® Blood and Tissue Kit (QIAGEN, Hilden, Germany) according to the manufacturer’s instructions. The amount of chlamydial DNA in all vPCR and nanopore sequencing samples was quantified with a sensitive *Chlamydiaceae* qPCR [47]. For subsequent nanopore sequencing of the DNA extracts (viable: 0.62 ng/µL; dead: 0.17 ng/µL), we followed the same approach as described for *E. coli* above. We used three barcodes for each sample, resulting in DNA input of 18.66 ng and 5.07 ng for the “viable” and “dead” library, respectively. After following the same

processing steps as established for the *E. coli* experiment, we filtered reads at a minimum average quality score of 8 and a minimum length of 100 b using Nanofilt v2.8.0 [54], and created *de novo* assemblies using metaFlye v2.9.1 [55], followed by polishing using minimap2 v2.17 [56] and three rounds of Racon v1.5 [57]. We finally used Kraken2 v2.0.7 [58] and the NCBI nt database for taxonomic classification of the assembled contigs.

Data Availability Statement

All raw data has been made publicly available via ENA (study accession number: PRJEB76420). All code has been made publicly available via Github: https://github.com/Genomics4OneHealth/Squiggle4Viability.git.

Financial Disclosure Statement

This study was funded by a Helmholtz Principal Investigator Grant awarded to LU. HU was supported by the Helmholtz Association under the joint research school “HIDSS-006 - Munich School for Data Science@Helmholtz, TUM&LMU”. EM was supported by an EASTBIO studentship, funded by BBSRC Grant Number BB/M010996/1, and an STFC Food Network+ Scoping Grant. SB and SK were supported by Helmholtz AI’s Helmholtz Association Initiative and Networking Fund. Computational Resources were provided by Helmholtz Munich and by the Helmholtz Association Initiative and Networking Fund HAICORE partition at the Forschungszentrum Jülich. LU, HU, and JMF have previously received travel and accommodation expenses to speak at Oxford Nanopore Technologies’ conferences.

Acknowledgments

We thank the laboratory of the Research Unit of Comparative Microbiome Analysis at Helmholtz Munich, Germany, especially Cornelia Galonska, for their support in processing the *Escherichia coli* samples. We thank the laboratory of the Institute of Veterinary Pathology, Switzerland, especially Theresa Pesch, for their support in processing the *Chlamydia abortus* samples. We further thank Valentin Rauscher for his help in training several deep models during his internship in the Urban research group at Helmholtz Munich.

Competing interests

The authors declare no competing interests.

References

1. Lewis WH, Tahon G, Geesink P, Sousa DZ, Ettema TJG. Innovations to culturing the uncultured microbial majority. Nat Rev Microbiol. 2021 Apr;19(4):225-40.
2. Lloyd KG, Steen AD, Ladau J, Yin J, Crosby L. Phylogenetically novel uncultured microbial cells dominate Earth microbiomes. mSystems. 2018 Sep 25;3(5).
3. Human Microbiome Jumpstart Reference Strains Consortium, Nelson KE, Weinstock GM, Highlander SK, Worley KC, Creasy HH, et al. A catalog of reference genomes from the human microbiome. Science. 2010 May 21;328(5981):994-9.
4. Sauerborn E, Corredor C, Reska T, Perlas A, Vargas da Fonseca Atum S, Goldman N, Wantia N, Prazeres da Costa C, Foster-Nyarko E, Urban L. Detection of hidden antibiotic resistance through real-time genomics. Preprint. Research Square. 2023 Dec 4. Available from: https://doi.org/10.21203/rs.3.rs-3620416/v1.
5. Handelsman J. Metagenomics: application of genomics to uncultured microorganisms. Microbiol Mol Biol Rev. 2004 Dec;68(4):669-85..
6. Pace NR. Mapping the tree of life: progress and prospects. Microbiol Mol Biol Rev. 2009 Dec;73(4):565-76.
7. Brooks JP, Edwards DJ, Harwich MD, Rivera MC, Fettweis JM, Serrano MG, et al. The truth about metagenomics: quantifying and counteracting bias in 16S rRNA studies. BMC Microbiol. 2015;15:66.
8. Dick GJ, Andersson AF, Baker BJ, Simmons SL, Thomas BC, Yelton AP, Banfield JF. Community-wide analysis of microbial genome sequence signatures. Genome Biol. 2009;10(8).
9. Quince C, Walker AW, Simpson JT, Loman NJ, Segata N. Shotgun metagenomics, from sampling to analysis. Nat Biotechnol. 2017 Sep;35(9):833-44.
10. Sereika M, Kirkegaard RH, Karst SM, Michaelsen TY, Sørensen EA, Wollenberg RD, Albertsen M. Oxford Nanopore R10.4 long-read sequencing enables the generation of near-finished bacterial genomes from pure cultures and metagenomes without short-read or reference polishing. Nat Methods. 2022 Jul;19(7):823-6.
11. Liu L, Yang Y, Deng Y, Zhang T. Nanopore long-read-only metagenomics enables complete and high-quality genome reconstruction from mock and complex metagenomes. Microbiome. 2022 Dec 2;10(1):209.
12. Jain M, Olsen HE, Paten B, Akeson M. The Oxford Nanopore MinION: delivery of nanopore sequencing to the genomics community. Genome Biol. 2016;17:239.
13. Pagès-Gallego M, de Ridder J. Comprehensive benchmark and architectural analysis of deep learning models for nanopore sequencing basecalling. Genome Biol. 2023 Apr 11;24(1):71.
14. Urban L, Perlas A, Francino O, Martí-Carreras J, Muga BA, Mwangi JW, et al. Real-time genomics for One Health. Mol Syst Biol. 2023 Aug 8;19(8).
15. Nogva HK, Drømtorp SM, Nissen H, Rudi K. Ethidium monoazide for DNA-based differentiation of viable and dead bacteria by 5'-nuclease PCR. Biotechniques. 2003 Apr;34(4):804-8, 810, 812-3.
16. Hellmann KT, Tuura CE, Fish J, Patel JM, Robinson DA. Viability-resolved metagenomics reveals antagonistic colonization dynamics of Staphylococcus epidermidis strains on preterm infant skin. mSphere. 2021 Oct 27;6(5).
17. Reska T, Pozdniakova S, Borras S, Schloter M, Cañas L, Perlas A, Rodó X, Winkler B, Schnitzler JP, Urban L. Air monitoring by nanopore sequencing. bioRxiv. 2023 Dec 19:2023.12.19.572325. doi:10.1101/2023.12.19.572325.
18. Morré SA, Sillekens PT, Jacobs MV, de Blok S, Ossewaarde JM, van Aarle P, et al. Monitoring of Chlamydia trachomatis infections after antibiotic treatment using RNA detection by nucleic acid sequence based amplification. Mol Pathol. 1998 Jun;51(3):149-54.
19. Herman L. Detection of viable and dead Listeria monocytogenes by PCR. Food Microbiol. 1997 Apr;14(2):103-110.
20. Min J, Baeumner AJ. Highly sensitive and specific detection of viable Escherichia coli in drinking water. Anal Biochem. 2002 Apr 15;303(2):186-93.
21. Urban L, Holzer A, Baronas JJ, Hall MB, Braeuninger-Weimer P, Scherm MJ, et al. Freshwater monitoring by nanopore sequencing. eLife. 2021 Jan 19;10.
22. Kumar SS, Ghosh AR. Assessment of bacterial viability: a comprehensive review on recent advances and challenges. Microbiology (Reading). 2019 Jun;165(6):593-610.
23. Taylor-Brown A, Madden D, Polkinghorne A. Culture-independent approaches to chlamydial genomics. Microb Genom. 2018 Feb;4(2).
24. Nocker A, Camper AK. Novel approaches toward preferential detection of viable cells using nucleic acid amplification techniques. FEMS Microbiol Lett. 2009 Feb;291(2):137-42.

25. Sheridan GE, Masters CI, Shallcross JA, Mackey BM. Detection of mRNA by reverse transcription-PCR as an indicator of viability in Escherichia coli cells. Appl Environ Microbiol. 1998 Apr;64(4):1313-8.
26. Wong VY, Duval MX. Inter-laboratory variability in array-based RNA quantification methods. Genomics Insights. 2013 May 6;6:13-24.
27. Hønsvall BK, Robertson LJ. From research lab to standard environmental analysis tool: Will NASBA make the leap? Water Res. 2017 Feb 1;109:389-397.
28. Janssen KJH, Dirks JAMC, Dukers-Muijrers NHTM, Hoebe CJPA, Wolffs PFG. Review of Chlamydia trachomatis viability methods: assessing the clinical diagnostic impact of NAAT positive results. Expert Rev Mol Diagn. 2018 Aug;18(8):739-747.
29. Nocker A, Cheung CY, Camper AK. Comparison of propidium monoazide with ethidium monoazide for differentiation of live vs. dead bacteria by selective removal of DNA from dead cells. J Microbiol Methods. 2006 Nov;67(2):310-20.
30. Emerson JB, Adams RI, Román CMB, Brooks B, Coil DA, Dahlhausen K, et al. Schrödinger's microbes: Tools for distinguishing the living from the dead in microbial ecosystems. Microbiome. 2017 Aug 16;5(1):86.
31. Bae S, Wuertz S. Discrimination of viable and dead fecal Bacteroidales bacteria by quantitative PCR with propidium monoazide. Appl Environ Microbiol. 2009 May;75(9):2940-4.
32. Courcelle J, Donaldson JR, Chow KH, Courcelle CT. DNA damage-induced replication fork regression and processing in Escherichia coli. Science. 2003 Feb 14;299(5609):1064-7.
33. Setlow RB, Swenson PA, Carrier WL. Thymine dimers and inhibition of DNA synthesis by ultraviolet irradiation of cells. Science. 1963 Dec 13;142(3598):1464-6.
34. Shibai A, Takahashi Y, Ishizawa Y, Motooka D, Nakamura S, Ying BW, Tsuru S. Mutation accumulation under UV radiation in Escherichia coli. Sci Rep. 2017 Nov 6;7(1):14531.
35. Ni P, Huang N, Zhang Z, Wang DP, Liang F, Miao Y, Xiao CL, Luo F, Wang J. DeepSignal: detecting DNA methylation state from Nanopore sequencing reads using deep-learning. Bioinformatics. 2019 Nov 1;35(22):4586-95.
36. Stoiber M, Quick J, Egan R, Lee JE, Celniker S, Neely RK, Loman N, Pennacchio LA, Brown J. De novo identification of DNA modifications enabled by genome-guided nanopore signal processing. bioRxiv. 2016 Dec 15;094672. doi:10.1101/094672.
37. Wang X, Ahsan MU, Zhou Y, Wang K. Transformer-based DNA methylation detection on ionic signals from Oxford Nanopore sequencing data. Quant Biol. 2023;11(3):287-96.
38. An N, Fleming AM, White HS, Burrows CJ. Nanopore detection of 8-oxoguanine in the human telomere repeat sequence. ACS Nano. 2015 Apr 28;9(4):4296-307.
39. Turin L, Surini S, Wheelhouse N, Rocchi MS. Recent advances and public health implications for environmental exposure to Chlamydia abortus: from enzootic to zoonotic disease. Vet Res. 2022 May 31;53(1):37.
40. Zhou B, Khosla A, Lapedriza A, Oliva A, Torralba A. Learning deep features for discriminative localization. In: Proceedings of the IEEE Conference on Computer Vision and Pattern Recognition; 2016 Jun 27-30; Las Vegas, NV, USA. p. 2921-9.
41. Ferguson JM, Smith MA. SquiggleKit: a toolkit for manipulating nanopore signal data. Bioinformatics. 2019 Dec 15;35(24):5372-3.
42. Gamaarachchi H, Samarakoon H, Jenner SP, Ferguson JM, Amos TG, Hammond JM, et al. Fast nanopore sequencing data analysis with SLOW5. Nat Biotechnol. 2022 Jul;40(7):1026-9.
43. Borel N, Polkinghorne A, Pospischil A. A review on chlamydial diseases in animals: still a challenge for pathologists? Vet Pathol. 2018 May;55(3):374-90.
44. Borel N, Sachse K. Zoonotic transmission of Chlamydia spp.: known for 140 years, but still underestimated. In: Sing A, editor. Zoonoses: infections affecting humans and animals. Cham: Springer International Publishing; 2023. p. 1-28.
45. Borel N, Marti H, Pospischil A, Pesch T, Prähauser B, Wunderlin S, et al. Chlamydiae in human intestinal biopsy samples. Pathog Dis. 2018 Nov 1;76(8).
46. Janssen KJ, Hoebe CJ, Dukers-Muijrers NH, Eppings L, Lucchesi M, Wolffs PF. Viability-PCR shows that NAAT detects a high proportion of DNA from non-viable Chlamydia trachomatis. PLoS One. 2016 Nov 3;11(11).
47. Loehrer S, Hagenbuch F, Marti H, Pesch T, Hässig M, Borel N. Longitudinal study of Chlamydia pecorum in a healthy Swiss cattle population. PLoS One. 2023 Dec 11;18(12).
48. McDonald MD, Owusu-Ansah C, Ellenbogen JB, Malone ZD, Ricketts MP, Frolking SE, et al. What is microbial dormancy? Trends Microbiol. 2024 Feb;32(2):142-50.

49. Potgieter M, Bester J, Kell DB, Pretorius E. The dormant blood microbiome in chronic, inflammatory diseases. FEMS Microbiol Rev. 2015 Jul;39(4):567-91.
50. Borel N, Dumrese C, Ziegler U, Schifferli A, Kaiser C, Pospischil A. Mixed infections with Chlamydia and porcine epidemic diarrhea virus - a new in vitro model of chlamydial persistence. BMC Microbiol. 2010 Jul 27;10:201.
51. Howard L, Orenstein NS, King NW. Purification on renografin density gradients of Chlamydia trachomatis grown in the yolk sac of eggs. Appl Microbiol. 1974 Jan;27(1):102-6.
52. Marti H, Biggel M, Shima K, Onorini D, Rupp J, Charette SJ, Borel N. Chlamydia suis displays high transformation capacity with complete cloning vector integration into the chromosomal rrn-nqrF plasticity zone. Microbiol Spectr. 2023 Dec 12;11(6).
53. Leonard CA, Schoborg RV, Borel N. Damage/danger associated molecular patterns (DAMPs) modulate Chlamydia pecorum and C. trachomatis serovar E inclusion development in vitro. PLoS One. 2015 Aug 6;10(8).
54. De Coster W, D'Hert S, Schultz DT, Cruts M, Van Broeckhoven C. NanoPack: visualizing and processing long-read sequencing data. Bioinformatics. 2018 Aug 1;34(15):2666-9.
55. Kolmogorov M, Bickhart DM, Behsaz B, Gurevich A, Rayko M, Shin SB, Kuhn K, Yuan J, Polevikov E, Smith TPL, Pevzner PA. metaFlye: scalable long-read metagenome assembly using repeat graphs. Nat Methods. 2020 Nov;17(11):1103-10.
56. Li H. Minimap2: pairwise alignment for nucleotide sequences. Bioinformatics. 2018 Sep 15;34(18):3094-100.
57. Vaser R, Sović I, Nagarajan N, Šikić M. Fast and accurate de novo genome assembly from long uncorrected reads. Genome Res. 2017 May;27(5):737-46.
58. Wood DE, Lu J, Langmead B. Improved metagenomic analysis with Kraken 2. Genome Biol. 2019 Nov 28;20(1):257.

Supporting information

Table S1. Performance Metrics of all Deep Neural Network Architectures tested for Viabiliy Inferences. Test set performance of residual neural network (ResNet) and transformer architectures, trained on various data modalities (Nanopore “Signal” or DNA sequence aka “Nucleotide”) and various signal chunk sizes (LR=1e-4).

Model Architecture	Data Modality	Length [Signal/Base]	Accuracy	F1	Precision	Sensitivity	Specificity	AUROC	AUPR
ResNet1	Signal	10K	0.83	0.84	0.78	0.91	0.74	0.90	0.87
ResNet2	Signal	10K	0.83	0.85	0.77	0.94	0.72	0.89	0.86
ResNet3	Signal	10K	0.81	0.82	0.76	0.89	0.72	0.87	0.83
Transformer	Signal	10K	0.79	0.82	0.73	0.92	0.67	0.86	0.82
ResNet1	Nucleotide (A,C,G,T)	800	0.51	0.51	0.51	0.52	0.50	0.52	0.53
ResNet1	Nucleotide (A,C,G,T,M)	800	0.51	0.50	0.51	0.50	0.52	0.51	0.51
ResNet1	Signal	1K	0.58	0.65	0.55	0.80	0.35	0.61	0.58
ResNet1	Signal	5K	0.71	0.76	0.66	0.89	0.54	0.78	0.73
ResNet1	Signal	7K	0.77	0.79	0.72	0.88	0.65	0.83	0.79
ResNet1	Signal	12K	0.84	0.85	0.77	0.97	0.71	0.91	0.88
ResNet1	Signal	20K	0.89	0.90	0.85	0.95	0.83	0.94	0.91

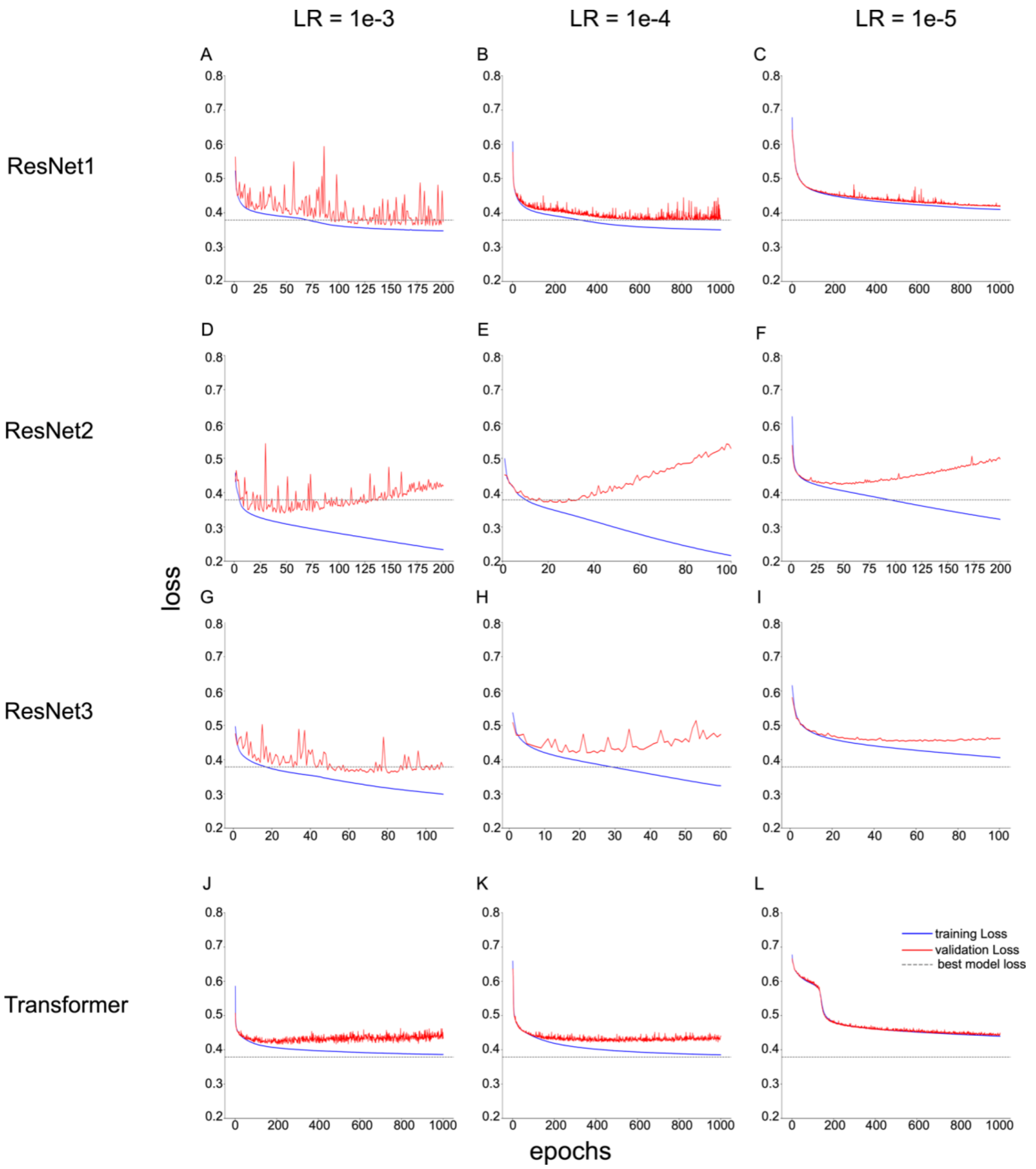

Fig S1. Training and Validation Loss across Deep Neural Network Architectures tested for Nanopore-Signal based Viability Inference, and across different Learning Rates.

(A-C) Model loss of ResNet1 at LRs of 1e-3, 1e-4, and 1e-5; (D-F) Model loss of ResNet2 at LRs of 1e-3, 1e-4, and 1e-5; (G-I) Model loss of ResNet3 at LRs of 1e-3, 1e-4, and 1e-5; and (J-L) Model loss of the transformer models at LRs of 1e-3, 1e-4, and 1e-5. The solid blue line indicates the training loss, the solid red line indicates the validation loss, and the dashed line indicates the minimum validation loss from the final ResNet1, LR=1e-4, model.

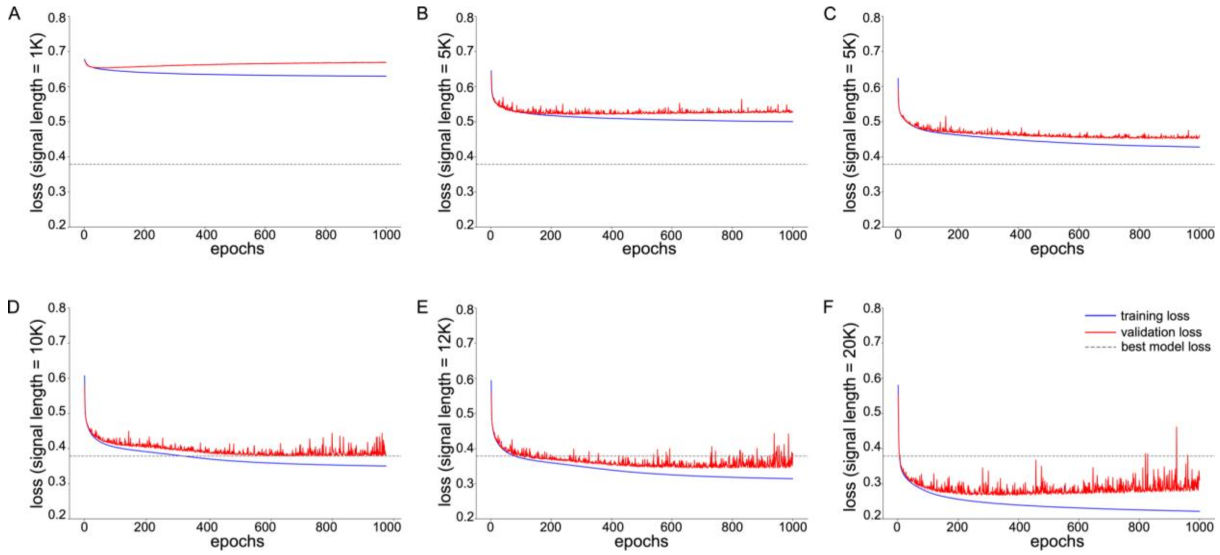

Fig S2. Training and Validation Loss of ResNet1 at various signal chunk sizes.
The signal chunk size varies from (A) 1k, (B) 5k, (C) 7k, (D) 10k, to (E) 12k and (F) 20k. The solid blue line indicates the training loss, the solid red line indicates the validation loss, and the dashed line indicates the minimum validation loss from the final (D) ResNet1model using a signal chunk size of 10k.

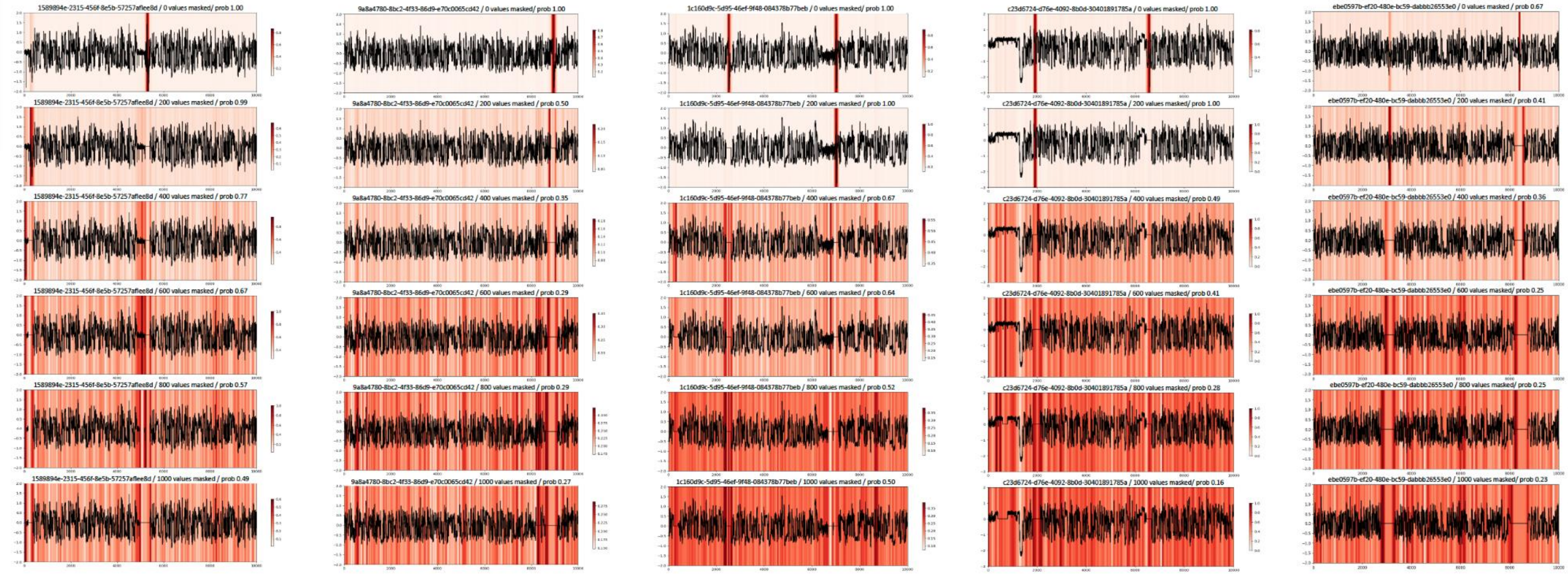

Fig S3. Exemplary drops in ResNet1 prediction probabilities in nanopore signal chunks after consecutive masking of the signal region with the respectively highest CAM value. *Figure headers*: signal chunk ID / total number of masked signal values / prediction probability per signal chunk “prob”. *Left to right*: Five exemplary nanopore signal chunks (length of 10k signals). *Top to bottom*: Consecutive masking of 200 signal values per masking event (Materials and Methods). *Legends*: Red-colored CAM value vizualisations.

Click here to access/download;Figure;Fig1.pdf

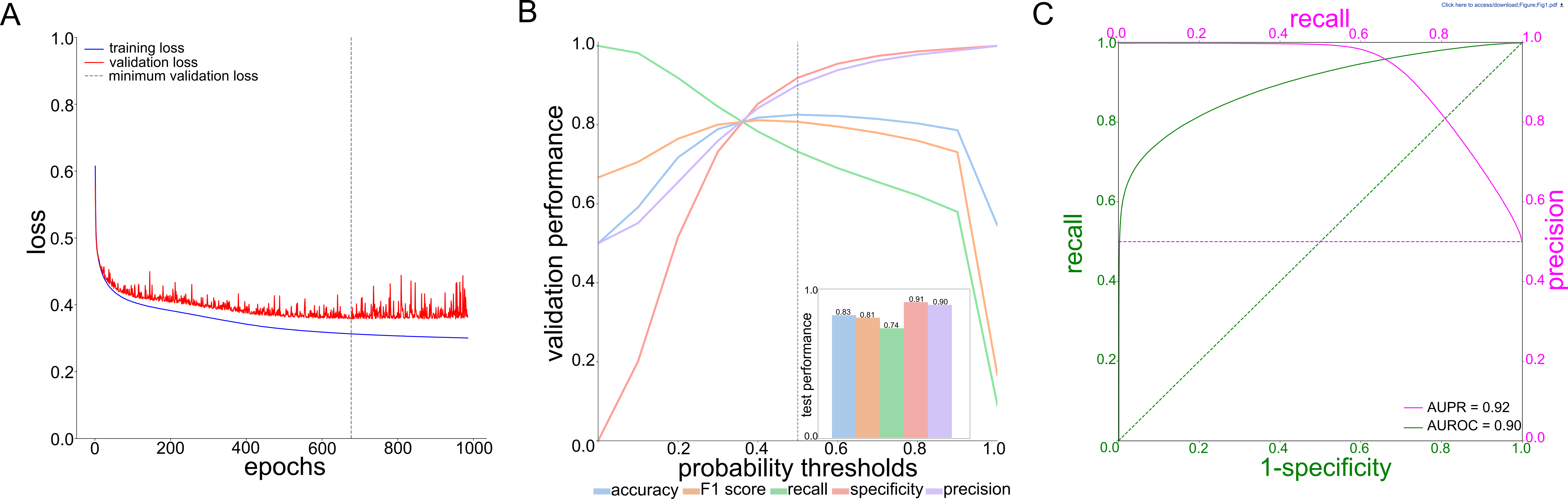

Click here to access/download;Figure;Fig2.pdf

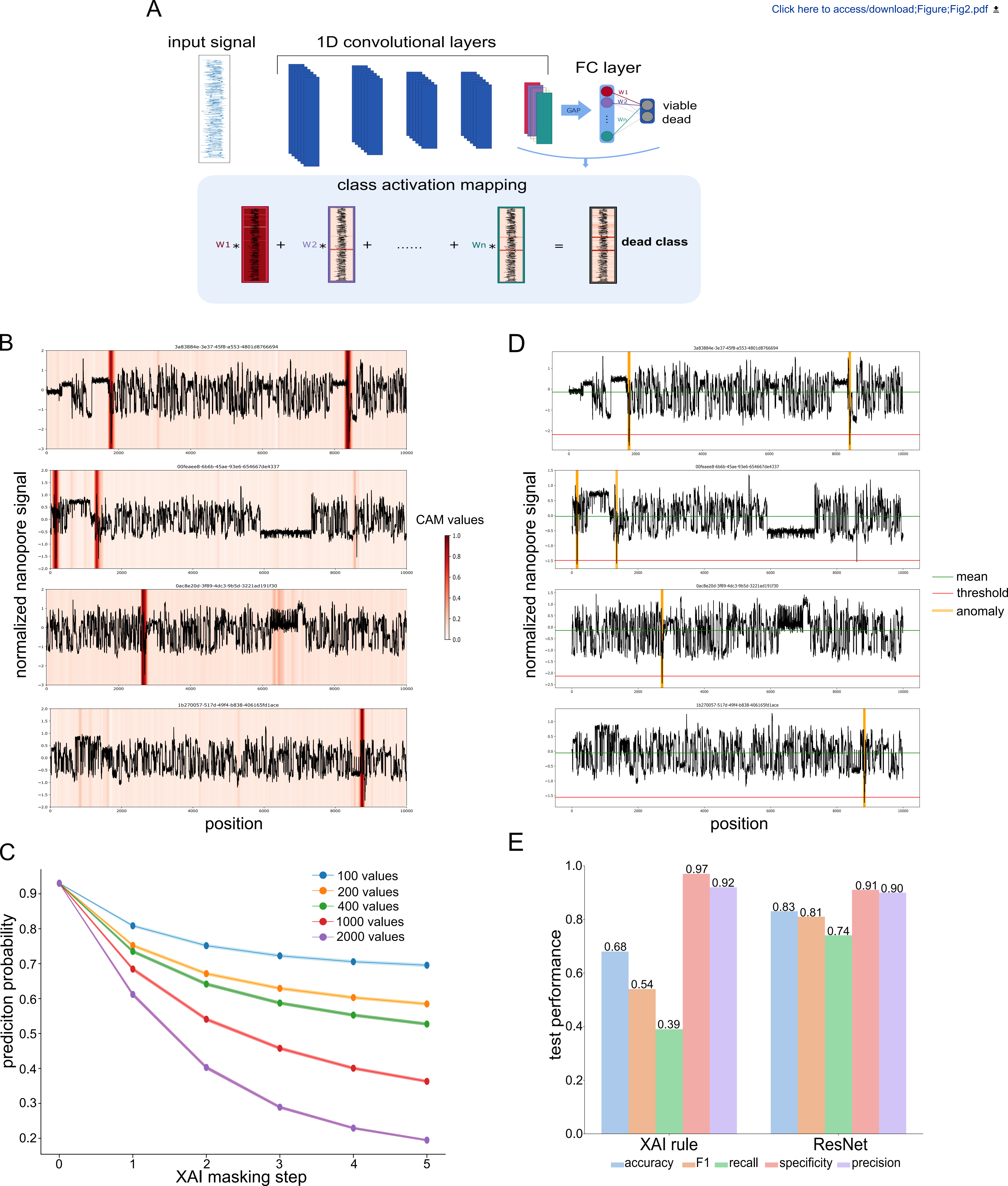

Click here to access/download;Figure;Fig3.pdf

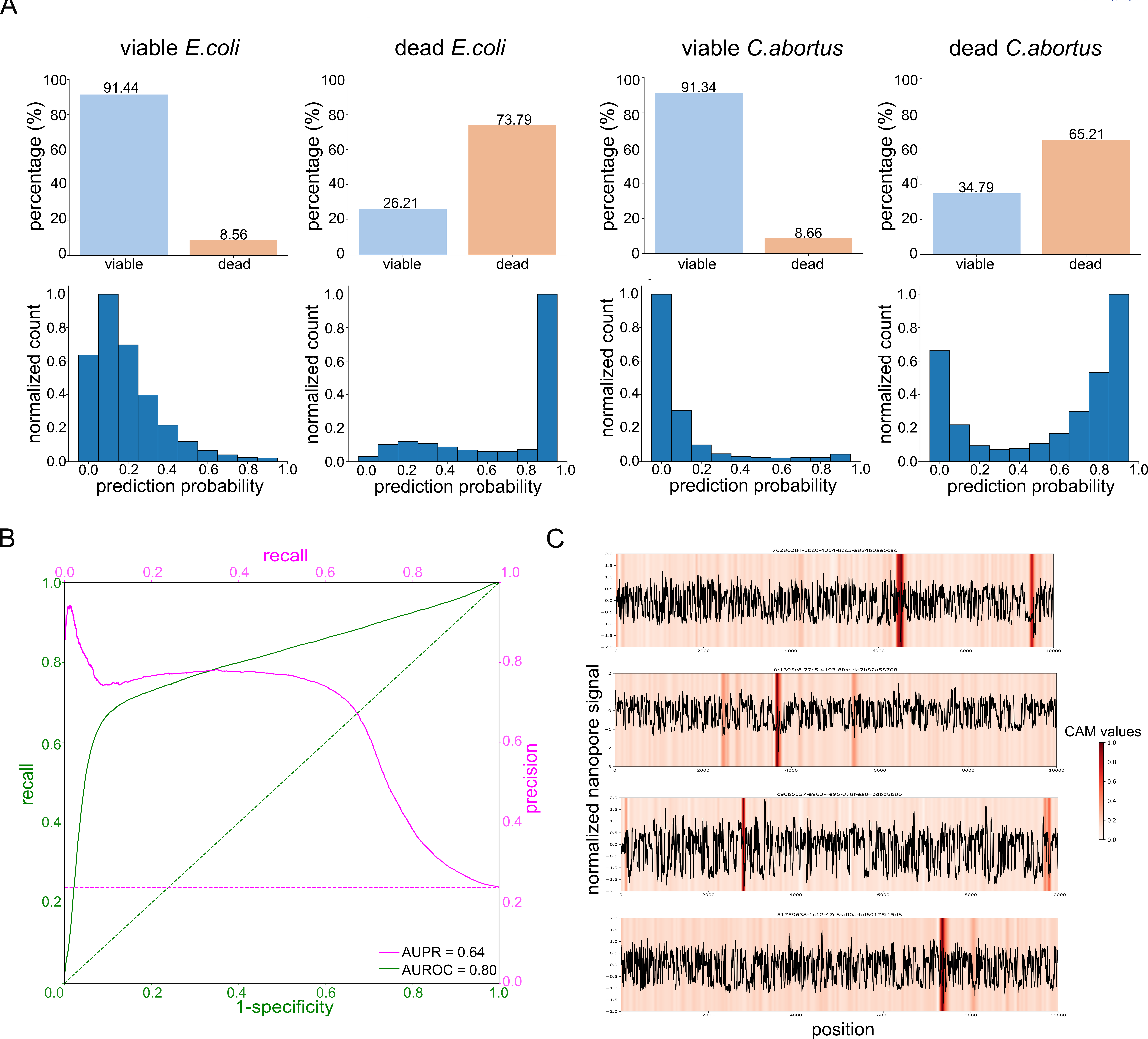

Click here to access/download;Figure;FigS1.pdf

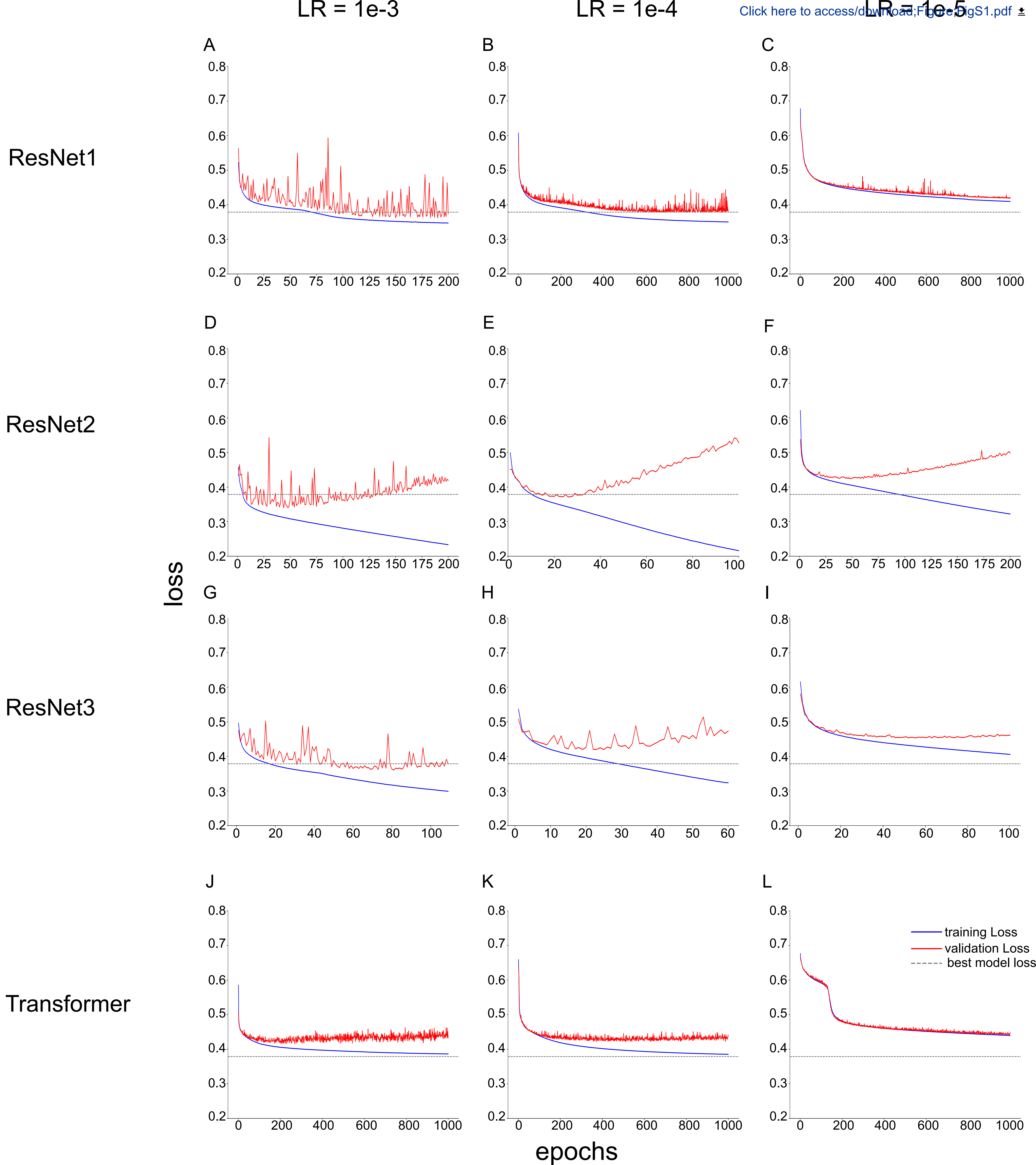

Click here to access/download;Figure;FigS2.pdf

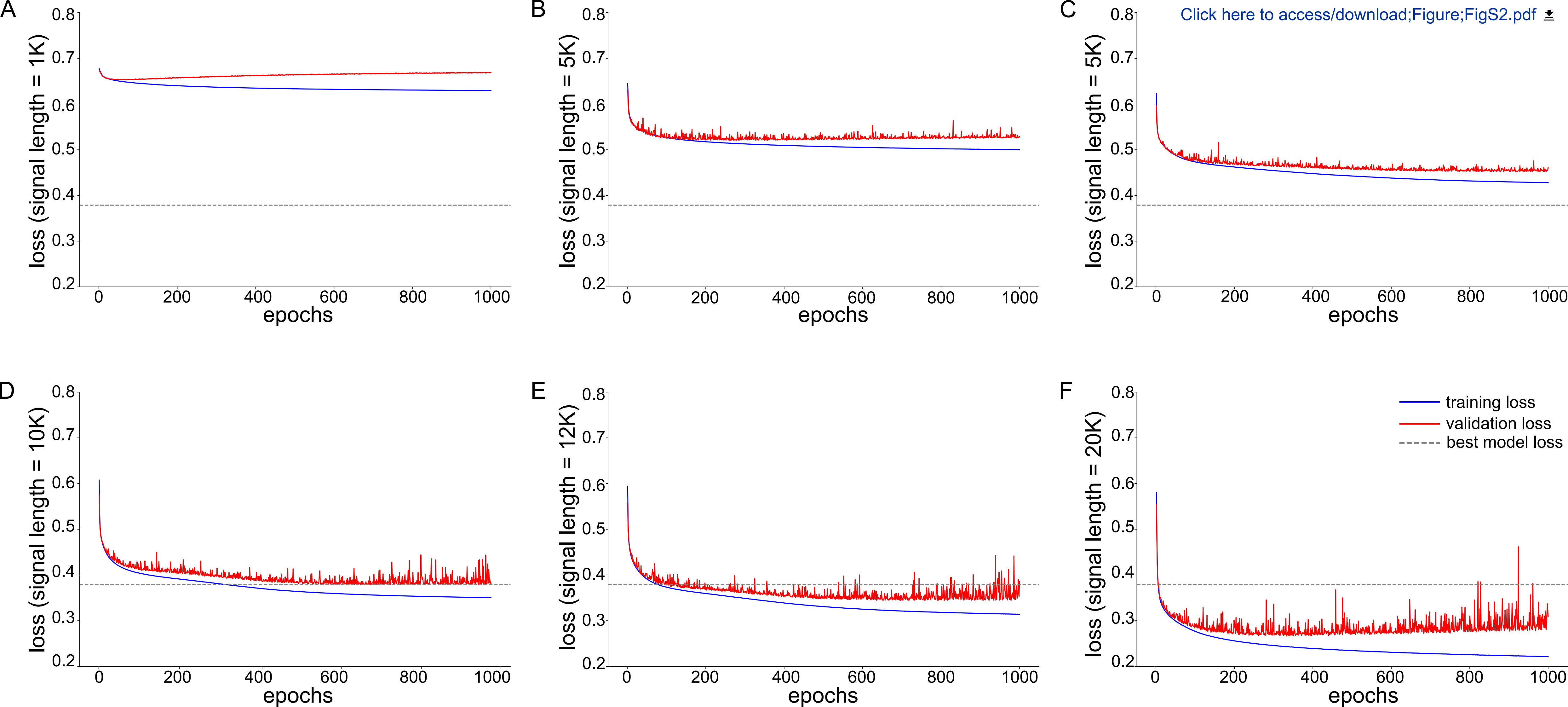

Click here to access/download;Figure;FigS3.pdf

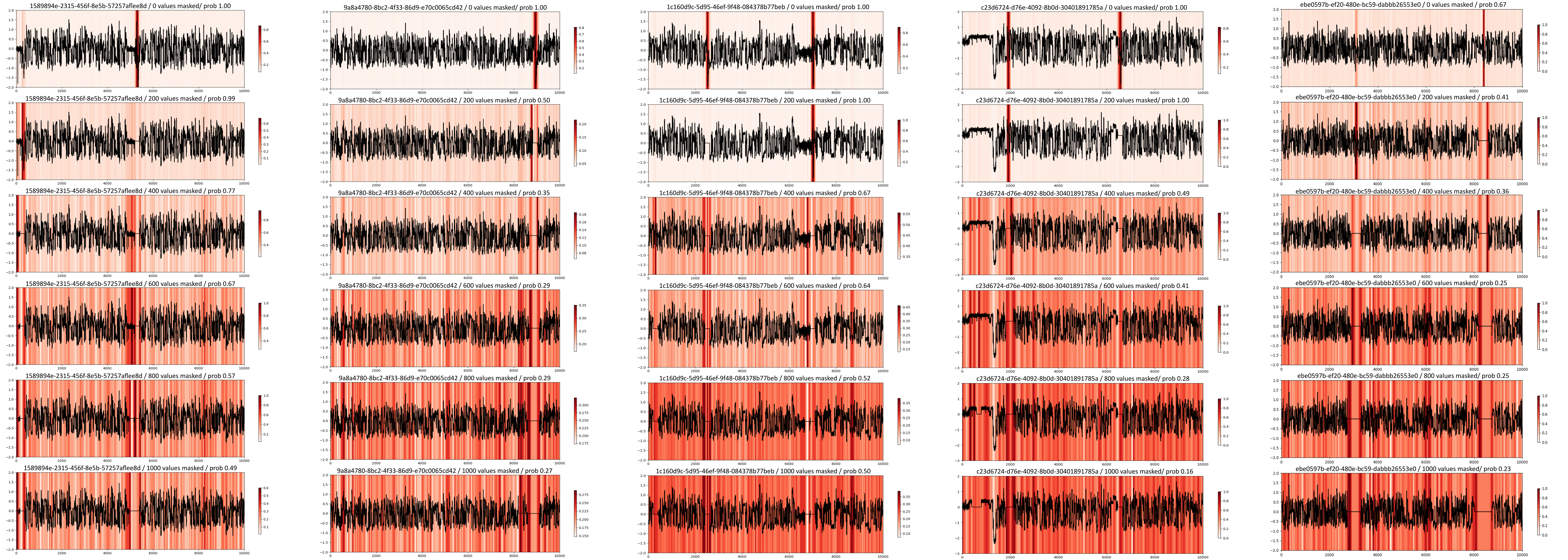

Click here to access/download;Personal Cover;cover_letter_GigaScience.pdf

Lara Urban
Principal Investigator Helmholtz Munich & Technical University of Munich
lara.h.urban@gmail.com; +49 (0) 160 92 105 701

Munich, September 6th 2024

Dear members of the GigaScience Editorial Board,

I would herewith like to submit our manuscript "**Nanopore- and AI-empowered metagenomic viability inference**" for peer review by GigaScience.

While metagenomic approaches, especially in combination with long-read sequencing technology, have provided a wealth of previously inaccessible information on microbial diversity, including *de novo* taxonomic detections and functional inferences, they have suffered from their inherent deficiency of not being able to differentiate between viable and dead microorganisms. Viability-resolved metagenomics might, however, be crucial for various microbial inferences, ranging from assessing ecosystem functions of environmental microbiomes to inferring the virulence of potential pathogens. While established viability-resolved metagenomic approaches are labor-intensive as well as biased and lacking in sensitivity, we here introduce a **new fully computational framework that leverages nanopore sequencing technology to assess microbial viability directly from freely available nanopore signal data**. We utilize deep neural networks to learn features from raw nanopore signals that can distinguish DNA from viable and dead microorganisms in a controlled experimental setting. The application of explainable AI tools then allows us to robustly pinpoint the signal patterns in the nanopore signals that allow the model to make viability predictions at high accuracy.

We importantly show that our **framework can be leveraged in a real-world application to estimate the viability of pathogenic *Chlamydia***, where traditional culture-based methods suffer from inherently high false negative rates. While this application shows that our viability model captures predictive patterns in the nanopore signals that can in principle be utilized to predict viability across taxonomic boundaries and independent of the killing method used to induce bacterial cell death, we emphasize that the generalizability of our computational framework needs to be assessed in much more detail in the future. Nevertheless, we here demonstrate for the first time the potential of analyzing freely available nanopore signal data to infer the viability of microorganisms, with many applications in environmental, veterinary, and clinical settings.

Our fully computational framework holds the promise of quantifying the impact of dead microorganisms in metagenomic studies by leveraging all existing and future nanopore metagenomic datasets, and to further explore factors such as species- and environment-specificity. Future quantitative modeling of viability further has the potential to inform more differentiated viability assessments, which might help quantify or even time degradation events and decipher the frequently discussed impact of dormancy on metagenomic studies. **Given the interdisciplinary nature of our study with a focus on establishing a fully computational framework based on the latest advances in (explainable) AI and nanopore technology to better understand basic principles of microbial biology and epidemiology, we believe that GigaScience would provide the perfect platform for peer review of our manuscript.** The open and transparent peer review and publishing standards supported by GigaScience further perfectly reflect the goal of our study to make computational approaches freely accessible to maximize the interpretability of existing data.

As potential reviewers we can recommend George Bouras @george.bouras@adelaide.edu.au and/or Ryan Wick @ryan.wick@unimelb.edu.au, Matthew Loose @matt.loose@nottingham.ac.uk, or Christopher Laumer @christopher.laumer1@nhm.ac.uk.

Thank you for your time, and we look forward to hearing from you.

Yours sincerely,

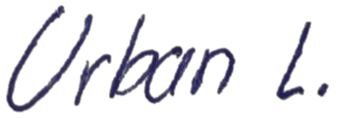

Supplement: giaf100_GIGA-D-24-00390_original_submission [file giaf100_giga-d-24-00390_original_submission.pdf]
