# Supplementary material for: Nanopore- and AI-empowered microbial viability inference
Source: Gigascience. 2025 Sep 3;14:giaf100. doi: 10.1093/gigascience/giaf100 (PMC12405693; doi:10.1093/gigascience/giaf100)
Supplement: giaf100_Authors_Response_To_Reviewer_Comments_original_submission [file giaf100_authors_response_to_reviewer_comments_original_submission.pdf]

Author's Response To Reviewer Comments

Dear Hans, dear GigaScience Editorial Office, dear reviewers,

We again thank you very much for giving us enough time to substantially revise our manuscript upon the helpful peer review we received from GigaScience. As discussed in our last communications with you and the reviewers, we corrected a major mistake on our side, and generated more data and ran several additional analyses. Thank you once more to the reviewers for their very helpful and insightful comments, which avoided the publication of wrong results.

Briefly, the reviewers' suggestions to move from a chunk- to a sequencing read-level analysis and to check the Chlamydia abortus dataset for any contaminating reads made us realize that the majority of chunks correctly classified as "dead" by our viabilty model stemmed from a few particularly long reads which were annotated as "unclassified" by taxonomic assignment. We in general realized that we had nearly no C. abortus reads in our "dead" dataset, which might have come from the strong heat exposure used as a killing method, and the resulting fragmentation of the C. abortus DNA. We are now glad that completely newly generated data on UV-killed Chlamydia suis viability shows that we can claim some generalizability of our viability predictions across taxonomic boundaries, while additional data on mock community viability after antibiotic exposure shows the limitations of our current model. In all newly generated data, we have further consistently included completely independent biological replicates. The analyses on the sequencing read-level have further substantially improved our model's prediction accuracies.

To make this peer review as comprehensive as possible, we here still keep relevant responses to the original reviewers' comments, and we additionally add details on the newly generated data and the new analysis results. All relevant changes in the manuscript are highlighted in yellow. I additionally respond to your major concerns here, and please find more details in the 1:1 responses to the reviewers' comments.

- Reviewer #1 points out that " the actual experiments and method developed are entirely based around sequencing of cultured clonal bacterial isolates", and therefore it is hard to say how the method performs with actual metagenomic data, which is more heterogeneous. This concern of the reviewer needs to be carefully addressed during the revision. [And: Echoing concerns of reviewer#1, reviewer #2 also wonder how well the results are generalizable.]

Thank you for these important comments. We toned down several paragraphs of our manuscript that relate to metagenomic applications; the new suggested title of the manuscript is "Nanopore- and AI-empowered microbial viability inference". We nevertheless argue that our computational framework has the potential for viability inferences in metagenomic data in the future: Our new data and analyses show that our viability model captures predictive patterns in the nanopore signal that can be utilized to predict viability across taxonomic boundaries; we, however, also show the limits of our model's generalizability through antibiotic exposure of a simple mock microbial community, where a new killing method-specific model has to be trained to obtain accurate viability predictions.

- Reviewer #1 also wonders whether a "much simpler logistic model using basic features such as read length and/or translocation could perform equivalently."

We have now included viability inference analyses based on read length and translocation speed in the manuscript, which is especially relevant now that we have moved to sequencing read-based viability inference. For read length, a simple logistic regression model resulted in a test set accuracy of only 0.50, an AUROC of 0.56, and an AUPR of 0.56, showing that read length is not a strong predictive feature. For translocation speed, a simple logistic regression model resulted in a test set accuracy of 0.59, an AUROC of 0.63, and an AUPR of 0.59, showing that translocation speed is also not a strong predictive feature of viability. Please see the 1:1 responses for more details.

- Another important issue is that the reviewer is concerned about potential data leakage between training and test set.

We ensured that no data leakage between training, validation, and test data was possible by first splitting our data on the sequencing read level and then subsequently chunking them, ensuring that each read was fully contained in only either of the three datasets. We describe this in more detail in our 1:1 responses. We now additionally generated data of completely independent biological replicates, taxonomic groups, and killing methods.

- Reviewer #2 recommends to provide the model in an easy-to-deploy form, and that's indeed something we highly encourage at GigaScience.

The UV ResNet1 (“UV_ecoli_ResNet_677ep.ckpt”) and antibiotic ResNet1 (“antibiotic_ecoli_ResNet_550ep.ckpt”) models are now available on our GitHub repository https://github.com/Genomics4OneHealth/Squiggle4Viability.git.

- Reviewer #2 couldn't locate the actual runs and pod5 files. I thought I have seen those on submission, but please double check these are indeed available and include instructions in the manuscript how to locate the raw data, including the pod5 files.

The pod5 data is indeed available via ENA; the previous version of the manuscript stated “All raw data has been made publicly available via ENA (study accession number: PRJEB76420).” We now additionally included the direct link to the pod5 files in the manuscript: https://www.ebi.ac.uk/ena/browser/view/PRJEB76420.

Thank you again very much for your time and patience. We look forward to receiving your feedback.

Best wishes, on behalf of all co-authors,
Lara

Reviewer #1

In this paper the authors train a ResNet-based model to predict whether individual 10,000 sample chunks of nanopore signal data originate from live or killed bacterial isolate cultures. From live and UV-killed (at exponential phase) E. coli K-12 cultures DNA was extracted and sequenced using separate R10.4.1 flowcells on a MinION. Signal data from each read in the live and dead extractions were then processed by discarding the first 1,500 samples and dividing the remaining signals into 10,000 sample chunks. These were then split into a balanced 60:20:20 train, test, and validation datasets with the constraint that no two chunks from the same read would end up in the same dataset (e.g., chunk 1 and chunk 2 of 1st read in the killed culture would hypothetically be separated into train and test). During this they also explored/compared the impact of chunk size, model architecture, and performance of a sequence based model using the E. coli data. With a nicely performed class-activation map and masking approach they then identified the signal regions most strongly associated with dead-predictions (such as twisting/kinking/pore blockage of DNA around pyrimidine dimers). Finally, they applied their trained model to a live and heat-killed Chlamydia abortus culture and compared their results to stained microscopy and propidium monoazide PCR measures of viability. They found equivalent performance on the C. abortus data to their E. coli data (despite a different killing-method and taxa).

The manuscript is well written and the methods are clearly described (including well documented code and deposited data). The authors explainability methodology is excellent although it would have been nice to see a bit more in-depth interpretation of those results. The authors have also presented a convincing case that nanopore signal data does contain information that can be used to distinguish signal chunks from live and dead bacterial monocultures. This methods has the potential to be useful in clinical and environmental genomics if it can be extended to more heterogeneous metagenomic samples. However, despite the title and framing of this manuscript (i.e., "metagenomics"), their analyses do not involve any metagenomic data

and their results so far do not demonstrate if this is fesible. Currently, the overall framing (and title) of the manuscript is not appropriate given the work performed at this point. Similarly, given that both E. coli and C. abortus "dead" cultures
resulted in median read length less than half the live cultures, the authors do not fully make the case that the signal and ResNet approach is actually required relative to simpler baseline models. Finally, although they did evaluate performance on a complete separate dataset, the authors should at least explore/quantify the correlation of live/dead prediction across chunks of the same read given the default expectation of non-independence of signal chunks from the same read.

We would very much like to thank the reviewer for their very helpful and insightful comments, which have substantially improved our manuscript and importantly avoided the publication of wrong results. In addition to the explanations sent to the editorial board above, we clarify a few questions/comments in more detail here.

Major
- Although the title and framing of the paper suggest that the authors are classifying live and dead bacteria in metagenomic datasets, the actual experiments and method developed are entirely based around sequencing of cultured clonal bacterial isolates. Metagenomic datasets are going to have considerably more heterogeneity in viability, species composition, and DNA signal characteristics. Given this, the paper's title, introduction, and parts of the discussion are a bit of an oversell and inappropriate. This manuscript should be revised to more clearly reflect the work actually performed.
Thank you for these important comments. We toned down several paragraphs of our manuscript that relate to metagenomic applications; the new suggested title of the manuscript is “Nanopore- and AI-empowered microbial viability inference”.
See, for example, the Discussion: pp. 13-14, ll. 482-516
We nevertheless argue that our computational framework has the potential for viability inferences in metagenomic data in the future: Our new data and analyses show that our viability model captures predictive patterns in the nanopore signal that can be utilized to predict viability across taxonomic boundaries; we specifically show that a model trained on UV-killed E. coli can also be applied to predict viability in UV-exposed Chlamydia suis, where established cultivation- and vPCR-based approaches for viability estimations are complicated due to the bacteria’s obligate intracellular life cycles.
See p. 10, ll. 373-393
We additionally show through explainable AI approaches that a similar pattern is detected by our model in both bacteria’s nanopore squiggle data. We, however, also show the limits of our model’s generalizability through antibiotic exposure of a simple mock microbial community of carbenicillin-susceptible E. coli and resistant Klebsiella oxytoca, where our UV-trained model incorrectly predicts similar levels of viability for both bacterial taxa. We then leverage the same residual neural network architecture of that model to train a new antibiotic viability model based on clean training data, and show its good performance on a held-out biological replicate as well as on the aforementioned mock community data.
See pp. 12-13, ll. 411-447
- This paper doesn't establish whether a ResNet + Signal approach actually outperforms a much simpler baseline. For example, given there is a clear extraction and median read-length differences between live and dead samples, it is possible that a much simpler logistic model using basic features such as read length and/or translocation could perform equivalently.
We now trained baseline models using the same UV-killed E. coli dataset that was used for training the residual neural network, and—as suggested—used simple logistic regression models with either sequencing read length or translocation speed as the only independent variable to predict binary viability as the dependent variable. For read length, this simple logistic regression resulted in a test set accuracy of only 0.50, an AUROC of 0.56, and an AUPR of 0.56, showing that read length is no or no strong predictive feature. For translocation speed, we first calculated the translocation speed as the number of basecalled bases per read divided by the sequencing time, where the time per read was estimated using the number of samples divided by the sampling rate. Here, the logistic regression resulted in a test set accuracy of 0.59, an AUROC of 0.63, and an AUPR of 0.59, showing that translocation speed is also not a strong predictive feature of viability. We added the results to the Table S1, and discuss them here:
See p. 9, ll. 330-345

- Although the C. abortus analysis demonstrates limited impact of leakage, I'm still a bit concerned that the

potential non-independence of chunks from the same read (i.e., chunk 1 and chunk 3 of the same read are more likely to share similar live/dead signal characteristics than Chunk 1 and 3 of different reads). By not having multiple chunks of the same read in the training, validation, or test datasets the authors may have avoided issues with longer-reads being more represented in their datasets. However, this has the potential to introduce data leakage between train and test set (which may impact generalisability when they attempt to extend this method to metagenomics). I think this paper would be improved by some exploration of the correlation of live/dead prediction across chunks of the same read. How often do different chunks of the same read disagree? How does this impact the overall performance of the model? Does taking the average prediction across chunks of the same read improve or degrade performance? Would this problem be better suited to a multiple instance learning approach (i.e., a live/dead label applied to all chunks from a single read) especially in more heterogeneous datasets? To what degree do longer reads with more chunks contribute disproportionately to the overall performance in the C. abortus dataset?
Thank you for raising these important points. First, we had previously ensured that all chunks from the same read were either included in the training, validation, or test dataset to avoid any data leakage between the datasets and to ensure that the reported test set performance is indeed based on sequencing reads that had never been seen by the model. Next, we now additionally explored in depth how our model performance (which was trained and validated based on a balanced set of chunks) would change if we move from a chunk- to a sequencing read-level-based analysis—which is also a lot more convenient for applications of our model in a genomic or metagenomic analysis which are obviously based on the sequencing read- and not on the chunk-level. While the chunk-based UV-killed E. coli test dataset was fully balanced with respect to the alive and dead categories, moving to the read level means that the same test dataset is not fully balanced anymore (due to some variability in read length distribution between the alive and dead classes) and comprised 59,171 viable reads and 72,207 dead E. coli reads.
We found that sequencing-read-based analysis substantially improved the performance of our model for viability inference in E. coli. First, we averaged the prediction probabilities of all chunks per sequencing read, which improved all the original chunk-based model performance parameters on the same held-out test dataset from 0.83 to 0.94 (accuracy), 0.81 to 0.94 (F1 score), 0.74 to 0.91 (recall), 0.91 to 0.97 (specificity), and from 0.90 to 0.97 (precision). This shows that chunks from the same sequencing read in general agree in terms of their prediction. Second, given that ResNets can make inferences on input data of varying signal length, we also obtained direct predictions for each full sequencing read, again using the same held-out test dataset. This "true" read-level analysis further improved classification performance in comparison to the read-level analysis that was based on averaging across chunks, namely from 0.94 to 0.96 (accuracy), 0.94 to 0.96 (F1 score), 0.91 to 0.94 (recall), and from 0.97 to 0.98 (precision). We incorporated only this final read-level analysis into the manuscript since it delivered superior results, and since it is easy to employ in any genomic or metagenomic application. As our previous chunk-based approach further required us to exclude any sequencing read shorter than 11,500 signals, we now additionally made read-level viability inferences on these short reads and included the results in the manuscript; we obtained good performance metrics, with an accuracy of 0.80, F1 of 0.78, precision of 0.84, recall of 0.73, specificity of 0.87, AUROC of 0.87 and AUPR of 0.89.
p. 9, ll. 330-345
While we agree that the Multiple Instance Learning framework would be another intriguing approach to create fixed-size embeddings from a consistent number of chunks during training while allowing for variable-sized embeddings for predictions to accommodate varying read lengths, we hope that our new ResNet-based inferences on sequencing reads of varying lengths are sufficient to show that read-level analyses not only work, but even substantially increase our performance.
Finally, we very much thank the reviewer for their suggestion to not only move from a chunk- to a sequencing read-level-based analysis, but also to check the Chlamydia abortus dataset for any contaminating reads, which, as they correctly state, can be present despite the de novo assembly of the sample resulting in a C. abortus genome. These analyses made us realize that the majority of C. abortus chunks that were correctly classified as dead stemmed from a few particularly long reads, which were annotated as "unclassified" by our subsequent Kraken2-based taxonomic assignment analyses (we hypothesize that these reads might have come from the eukaryotic host cells of this obligate intracellular parasite). We, in general, realized that we had nearly no C. abortus reads in our "dead" dataset, which might have come from the strong heat exposure used as a killing method, and the resulting fragmentation of the C. abortus DNA. We are now glad that completely newly generated data on UV-killed Chlamydia suis viability shows that we can claim some generalizability of our viability predictions across taxonomic boundaries, while additional data on mock community viability after antibiotic exposure shows the

limitations of our current model. In all newly generated data, we consistently included completely independent biological replicates, and further conducted all viability inferences on the sequencing read-level.
See p. 10, ll. 373-393
As we now do all inferences on the read-level now, this means that longer reads can not disproportionately contribute to the overall performance.
Minor

- SRA records don't seem to be live yet.

The data is available at https://www.ncbi.nlm.nih.gov/sra?linkname=bioproject_sra_all&from_uid=1123127. All necessary information is included in the manuscript.

- Are the actual pod5 files available?

The pod5 files are available at the following link: https://www.ebi.ac.uk/ena/browser/view/PRJEB76420. All necessary information is included in the manuscript.

- Read-level performance should be analysed and reported.
As discussed above, we conducted the read-level analyses and added them to the manuscript.
See p. 9, ll. 330-345

- Figure 1B: the test subplot numbers are almost too small to read - they may benefit from being its own panel.
We changed the Figure 1B's font size to make it more readable.
- Plot axes labels are not always clear (e.g., Figure 3) percentage of what? Chunks? or Reads? It would be nice to see consistent capitalisation of labels and legends.

The percentages represent the percentage of sequencing reads; we clarified this in the figure caption. We also made an effort to make the capitalization more consistent throughout the manuscript.

- Predictions on viable E. coli and viable C. abortus seems surprisingly similar (91.44% vs 91.34% viable and 8.56% vs 8.66% dead) despite different taxa, potentially underlying viable cell proportion, and output probability densities. This would benefit from further discussion/analysis - do misclassified chunks have any common characteristics? Would you expect the E. coli to have similar microscopy/PCR measured viability percentage as the C. abortus.

As already discussed, we removed the original C. abortus analyses from the manuscript, and we instead included more robust data on UV-killed C. suis. The percentage of correctly classified sequencing reads now differs more substantially between E. coli and C. suis (Fig. 3C).

- Would be good to see a bit more discussion/exploration of impact of mixed live/dead cells given ~37.6% viability measure in the C. abortus sample (e.g., how well do models perform with different ratios of live/dead reads) - could potentially be achieved using in-silico spike ins).

As already discussed, we removed the original C. abortus analyses from the manuscript. In the new more robust data on UV-killed C. suis, the viability measures are a lot clearer (e.g., only 3.85% of sequencing reads in the UV-exposed dataset ar3 classified as "viable"; Fig. 3C). We, however, fully agree that it will be important to assess if our model can accurately assess viability in mixed settings to then quantitatively predict viability per taxonomic group. We briefly discuss this in the manuscript (e.g., p. 14, ll. 500-505), and plan on including such spike-in experiments in our next big antibiotic exposure experiment.

Reviewer #2

Summary: Urel and colleagues present a novel computational method to predict viability from

metagenomic sequencing data, using the Nanopore squiggle as input. The manuscript is well-written and present an interesting new application, bolstered in particular by the application of explainable AI. However, I have some concerns regarding the generalizability of their method, detailed below.

We would very much like to thank the reviewer for their very helpful and insightful comments, which we believe substantially improved our manuscript and importantly avoided the publication of wrong results. In addition to the explanations sent to the editorial board, we clarify a few questions/comments in more detail here.

Major:
The way the authors try to exclude contamination in their C. abortus experiment is not optimal, since contaminatants might be at low abundance and therefore not assemble well (especially with the relatively low sequencing output overall). Instead, it would be better to map reads against the reference genome for C. abortus and check if reads predicted to be viable maps or if they are unmapped in this test. Maybe viable reads instead map against a database of known contaminants, like skin-resident microbes or other known kit contaminants. (This could potentially bolster their model performance).

We thank the reviewer very much for their suggestion to check the Chlamydia abortus dataset for any contaminating reads, which they correctly state can be present despite the de novo assembly of the sample resulting in a Chlamydia genome. We now used Kraken2 to obtain taxonomic classifications of all sequencing reads in the C. abortus dataset; we found that in the "viable" dataset, which contains 96,000 reads, only 13.30% (12,775 reads) were classified as belonging to the Chlamydia genus, and that in the "dead" dataset, which contains 74,639 reads, only 7.83% (5,845 reads) were classified as belonging to the Chlamydia genus. This analysis together with our sequencing read-level based viability inferences made us realize that the majority of Chlamydia chunks that were correctly classified as “dead” stemmed from a few particularly long reads, which were annotated as “unclassified” by Kraken 2-based taxonomic assignment. We therefore generated new Chlamydia data, this time from UV-killed Chlamydia suis to avoid strong fragmentation from heat exposure, and we can show that our viability model which was trained on UV-killed E. coli can predict viability in this C. suis dataset with high accuracy.

pp. 9-10, ll. 347-393

The authors claim that their method generalizes well from E. coli to C. abortus, which were killed in two different ways (UV and heat shock). However, if I understood correctly, their extracted DNA was left in the lab for 5 days. During this time, could exposure to sunlight over time have led to similar chemical reactions (meaning twists/kinks in the DNA as well as pyrmidine dimers)? This might be a point to discuss or it could be easily tested by incubating the DNA of the heat-killed C. abortus in the dark.
We thank Reviewer #2 for raising this important point, which we however believe might not be relevant anymore in its current form since we, as discussed above, removed the heat-exposed C. abortus data and instead added data on UV-exposed C. suis data to our manuscript. Using this experiment, we now only argue that our viability predictions can—to the extent tested—be generalized across taxonomic boundaries. With our additional data on mock community viability after antibiotic exposure, we instead show and discuss the limitations of our UV-trained viability model regarding generalizability across killing methods or sources of degradation.
pp. 12-13, ll. 410-448
What is the time-frame of DNA degradation in which the model works best? The authors left the DNA for 5 days, but metagenomic samples are usually processed quite quickly. How would the model perform on samples that were only kept for 1 day after initial killing? At which time of incubation does the model not generalize anymore? For a potential application, it might be useful to know if DNA is viable or not, even if the cells died relatively recently (and in the dark).
We thank the reviewer for raising these interesting suggestions. We would like to move our binary classifications to quantitative predictions in follow-up projects to explore if we can quantify the intensity and length of damage exposure; such data and analyses would answer several questions raised by the reviewer (see the Discussion, e.g., p. 14, ll. 500-505). In this first proof-of-concept study, we only focus on binary classifications and instead explore the more general question of generalizability of deep model-based viability predictions across taxa and killing methods. As we, however, generated new data from UV-exposed C. suis for this review, we had the chance to also sequence a C. suis sample directly after UV

exposure instead of adding additional incubation for five days as we did for the E. coli experiment, to at least answer the reviewer's specific question; we can therefore confirm that the UV ResNet can already predict viability with high accuracy directly after UV exposure (same accuracy of 0.93, same AUROC of 0.97, and AUPR of 0.94 of 0.95 before and after five-day incubation, respectively).
We further toned down several paragraphs of our manuscript that relate to metagenomic applications, and we now emphasize that our future goal is the application of our computational framework for viability inferences in highly heterogeneous metagenomic data (see, for example, the Discussion: pp. 13-14, ll. 482-516). I hope our manuscript now more clearly states that this study is a first exploration into whether nanopore squiggle signal encodes information that can theoretically be used for viability predictions. Our newly integrated data and analyses show that our viability model captures predictive patterns in the nanopore signal that can be utilized to predict viability across taxonomic boundaries; we, however, also show the limits of our model's generalizability through antibiotic exposure of a simple mock microbial community, where we take a first step towards metagenomic applications.
Code availability: The github looks great, but as a potential user of their method, I would not want to train my own model. Is it possible to host the model, maybe on Zenodo, so that it could be more useful as an application?

All code and the UV ResNet1 model ("UV_ecoli_ResNet_677ep.ckpt") and antibiotic ResNet1 model ("antibiotic_ecoli_ResNet_550ep.ckpt") have been made publicly available via Github: https://github.com/Genomics4OneHealth/Squiggle4Viability.git. We now specify this in the manuscript's Data Availability Statement (p. 9, ll. 708-712).

Minor:
Lines 96-100 read a bit like a Nanopore commercial and are not really relevant for this paper.

We removed these lines.

Line 182: shouldn't heat shock at 120 C inactivate enzymes?
We edited this statement in our manuscript to "In contrast, heat shock at 120°C and bead beating might not uniformly degrade all enzymatic activity [1, 2], potentially allowing residual DNA-degrading enzymes to persist and contribute to the degradation of genomic material during subsequent natural exposure." [p. 5, ll. 200-205], citing the two publications below. We are, however, also happy to remove this hypothesis since exposure to 120°C might have also just fragmented the DNA directly. Please let us know your preference.
[1] Wiame, I., Remy, S., Swennen, R., & Sági, L. (2000). Irreversible heat inactivation of DNase I without RNA degradation. BioTechniques, 29(2), 252–256.
[2] Hanaki, K., Nakatake, H., Yamamoto, K., Odawara, T., & Yoshikura, H. (2000). DNase I activity retained after heat inactivation in standard buffer. BioTechniques, 29(1), 38–42.

Line 206: it is curious to keep the default cutoff just because the results are fine. Why not optimize the F1 score, for example? Fig 1B seems to indicate that a probability threshold of 0.48 or something would give a higher F1 score. The decision to keep the threshold at the default value seems arbitrary.
We optimized the probability threshold based on the model's accuracy; we now clarify this in the manuscript (p. 5, ll. 225-226). We, however, agree that, based on the goal of the classification, this threshold could be adapted to optimize a different metric (e.g., specificity to ensure the detection of all viable pathogens).
Line 275: interesting hypothesis. Did you observe quicker decay of pore viability in the dead versus the alive run? Could you provide the pore scan information over the time of the sequencing run as a supplement, maybe, to back up this hypothesis?

Thank you for this interesting suggestion. We could unfortunately not compare pore decay between viable and UV-killed samples since we sequenced them on the same flow cell. We did, however, observe unusually quick pore decay when including any UV-killed samples on a flow cell, but we only have anecdotal evidence for this.

Line 311: the number does not match the one in the table

These results were removed.

Line 331: the dead reads are very short. Could you compare just the length of the reads with the viability predictions? Are shorter reads more likely to be predicted to be non-viable?

These results were removed. For the UV-exposed E. coli dataset, we however now included baseline viability inference just using sequencing read length as an input: A a simple logistic regression model resulted in a test set accuracy of only 0.50, an AUROC of 0.56, and an AUPR of 0.56, showing that read length is not a strong predictive feature.

pp. 6-7, ll. 262-276

Fig 3a: what does normalized count mean? How about a standard histogram or density plot?

The histograms (now Fig 3C; bottom row) are standard histograms, and only the y-axis scale is scaled to between 0 and 1 for better readability and comparability across datasets; we now however include the total number of reads in the figure caption to easily provide this information to the reader (59,171 "viable" and 72,207 "dead" E.coli sequencing reads, and 54,853 "viable" and 23,073 "dead" C. suis sequencing reads pooled across the two biological replicates; pp. 11-12, ll. 396-408).

Line 442: The most recent version of dorado is v0.8.2.; did you mean v0.4.2? Please adjust.

We here refer to the basecalling model 4.2.0 as, for example, described here: https://github.com/nanoporetech/dorado; we now specify this in the final manuscript (p. 15, ll. 542-544).

Editorial Manager®

Cookie Preference Center

We use cookies which are necessary to make our site work. We may also use additional cookies to analyze, improve and personalize our content and your digital experience. For more information, see our Cookie Policy.

You may choose not to allow some types of cookies. However, blocking some types may impact your experience of our site and the services we are able to offer. See the different category headings below to find out more or change your settings.

Allow all

Manage Consent Preferences

Strictly Necessary Cookies

Always active

These cookies are necessary for the website to function and cannot be switched off in our systems. They are usually only set in response to actions made by you which amount to a request for services, such as setting your privacy preferences, logging in or filling in forms. You can set your browser to block or alert you about these cookies, but some parts of the site will not then work.

Functional Cookies

These cookies enable the website to provide enhanced functionality and personalisation. They may be set by us or by third party providers whose services we have added to our pages. If you do not allow these cookies then some or all of these services may not function properly.

Performance Cookies

These cookies allow us to count visits and traffic sources so we can measure and improve the performance of our site. They help us to know which pages are the most and least popular and see how visitors move around the site.

Targeting Cookies

These cookies may be set through our site by our advertising partners. They may be used by those companies to build a profile of your interests and show you relevant adverts on other sites. If you do not allow these cookies, you will experience less targeted advertising.

Cookie List

Clear

Apply Cancel

Consent Leg.Interest

Confirm my choices

Powered by onetrust
