# Supplementary material for: Nanopore- and AI-empowered microbial viability inference
Source: Gigascience. 2025 Sep 3;14:giaf100. doi: 10.1093/gigascience/giaf100 (PMC12405693; doi:10.1093/gigascience/giaf100)
Supplement: giaf100_Reviewer_1_Report_Original_Submission — Finlay Maguire, Ph.D. -- 11/4/2024 [file giaf100_reviewer_1_report_original_submission.pdf]

Reviewer Report

Title: Nanopore- and AI-empowered microbial viability inference

Version: Original Submission **Date:** 11/4/2024

Reviewer name: Finlay Maguire, Ph.D.

Reviewer Comments to Author:

In this paper the authors train a ResNet-based model to predict whether individual 10,000 sample chunks of nanopore signal data originate from live or killed bacterial isolate cultures. From live and UV-killed (at exponential phase) E. coli K-12 cultures DNA was extracted and sequenced using separate R10.4.1 flowcells on a MinION. Signal data from each read in the live and dead extractions were then processed by discarding the first 1,500 samples and dividing the remaining signals into 10,000 sample chunks. These were then split into a balanced 60:20:20 train, test, and validation datasets with the constraint that no two chunks from the same read would end up in the same dataset (e.g., chunk 1 and chunk 2 of 1st read in the killed culture would hypothetically be separated into train and test). During this they also explored/compared the impact of chunk size, model architecture, and performance of a sequence based model using the E. coli data. With a nicely performed class-activation map and masking approach they then identified the signal regions most strongly associated with dead-predictions (such as twisting/kinking/pore blockage of DNA around pyrimidine dimers). Finally, they applied their trained model to a live and heat-killed Chlamydia abortus culture and compared their results to stained microscopy and propidium monoazide PCR measures of viability. They found equivalent performance on the C. abortus data to their E. coli data (despite a different killing-method and taxa).
The manuscript is well written and the methods are clearly described (including well documented code and deposited data). The authors explainability methodology is excellent although it would have been nice to see a bit more in-depth interpretation of those results. The authors have also presented a convincing case that nanopore signal data does contain information that can be used to distinguish signal chunks from live and dead bacterial monocultures. This methods has the potential to be useful in clinical and environmental genomics if it can be extended to more heterogeneous metagenomic samples. However, despite the title and framing of this manuscript (i.e., "metagenomics"), their analyses do not involve any metagenomic data and their results so far do not demonstrate if this is fesible. Currently, the overall framing (and title) of the manuscript is not appropriate given the work performed at this point. Similarly, given that both E. coli and C. abortus "dead" cultures resulted in median read length less than half the live cultures, the authors do not fully make the case that the signal and ResNet approach is actually required relative to simpler baseline models. Finally, although they did evaluate performance on a complete separate dataset, the authors should at least explore/quantify the correlation of live/dead prediction across chunks of the same read given the default expectation of non-independence of signal chunks from the same read.
Major
- Although the title and framing of the paper suggest that the authors are classifying live and dead bacteria in metagenomic datasets, the actual experiments and method developed are entirely based

around sequencing of cultured clonal bacterial isolates. Metagenomic datasets are going to have considerably more heterogeneity in viability, species composition, and DNA signal characteristics. Given this, the paper's title, introduction, and parts of the discussion are a bit of an oversell and inappropriate. This manuscript should be revised to more clearly reflect the work actually performed.
- This paper doesn't establish whether a ResNet + Signal approach actually outperforms a much simpler baseline. For example, given there is a clear extraction and median read-length differences between live and dead samples, it is possible that a much simpler logistic model using basic features such as read length and/or translocation could perform equivalently.
- Although the C. abortus analysis demonstrates limited impact of leakage, I'm still a bit concerned that the potential non-independence of chunks from the same read (i.e., chunk 1 and chunk 3 of the same read are more likely to share similar live/dead signal characteristics than Chunk 1 and 3 of different reads). By not having multiple chunks of the same read in the training, validation, or test datasets the authors may have avoided issues with longer-reads being more represented in their datasets. However, this has the potential to introduce data leakage between train and test set (which may impact generalisability when they attempt to extend this method to metagenomics). I think this paper would be improved by some exploration of the correlation of live/dead prediction across chunks of the same read. How often do different chunks of the same read disagree? How does this impact the overall performance of the model? Does taking the average prediction across chunks of the same read improve or degrade performance? Would this problem be better suited to a multiple instance learning approach (i.e., a live/dead label applied to all chunks from a single read) especially in more heterogeneous datasets? To what degree do longer reads with more chunks contribute disproportionately to the overall performance in the C. abortus dataset?

Minor

- SRA records don't seem to be live yet (https://www.ncbi.nlm.nih.gov/sra?linkname=bioproject_sra_all&from_uid=1123127)
- Are the actual pod5 files available?
- Read-level performance should be analysed and reported.
- Figure 1B: the test subplot numbers are almost too small to read - they may benefit from being its own panel.
- Plot axes labels are not always clear (e.g., Figure 3) percentage of what? Chunks? or Reads? It would be nice to see consistent capitalisation of labels and legends.
- Predictions on viable E. coli and viable C. abortus seems surprisingly similar (91.44% vs 91.34% viable and 8.56% vs 8.66% dead) despite different taxa, potentially underlying viable cell proportion, and output probability densities. This would benefit from further discussion/analysis - do misclassified chunks have any common characteristics? Would you expect the E. coli to have similar microscopy/PCR measured viability percentage as the C. abortus.
- Would be good to see a bit more discussion/exploration of impact of mixed live/dead cells given ~37.6% viability measure in the C. abortus sample (e.g., how well do models perform with different ratios of live/dead reads) - could potentially be achieved using in-silico spike ins).

Methods

Are the methods appropriate to the aims of the study, are they well described, and are necessary controls included? Choose an item.

Conclusions

Are the conclusions adequately supported by the data shown? Choose an item.

Reporting Standards

Does the manuscript adhere to the journal's guidelines on minimum standards of reporting? Choose an item.

Choose an item.

Statistics

Are you able to assess all statistics in the manuscript, including the appropriateness of statistical tests used? Choose an item.

Quality of Written English

Please indicate the quality of language in the manuscript: Choose an item.

Declaration of Competing Interests

Please complete a declaration of competing interests, considering the following questions:

- Have you in the past five years received reimbursements, fees, funding, or salary from an organisation that may in any way gain or lose financially from the publication of this manuscript, either now or in the future?
- Do you hold any stocks or shares in an organisation that may in any way gain or lose financially from the publication of this manuscript, either now or in the future?
- Do you hold or are you currently applying for any patents relating to the content of the manuscript?
- Have you received reimbursements, fees, funding, or salary from an organization that holds or has applied for patents relating to the content of the manuscript?
- Do you have any other financial competing interests?
- Do you have any non-financial competing interests in relation to this paper?

If you can answer no to all of the above, write 'I declare that I have no competing interests' below. If your reply is yes to any, please give details below.

I declare that I have no competing interests

I agree to the open peer review policy of the journal. I understand that my name will be included on my report to the authors and, if the manuscript is accepted for publication, my named report including any attachments I upload will be posted on the website along with the authors' responses. I agree for my report to be made available under an Open Access Creative Commons CC-BY license (http://creativecommons.org/licenses/by/4.0/). I understand that any comments which I do not wish to be included in my named report can be included as confidential comments to the editors, which will not be published.

Choose an item.

To further support our reviewers, we have joined with Publons, where you can gain additional credit to further highlight your hard work (see: https://publons.com/journal/530/gigascience). On publication of this paper, your review will be automatically added to Publons, you can then choose whether or not to claim your Publons credit. I understand this statement.

Yes Choose an item.
