# Supplementary material for: Nanopore- and AI-empowered microbial viability inference
Source: Gigascience. 2025 Sep 3;14:giaf100. doi: 10.1093/gigascience/giaf100 (PMC12405693; doi:10.1093/gigascience/giaf100)
Supplement: giaf100_Reviewer_2_Report_Original_Submission — Jakob Wirbel -- 11/5/2024 [file giaf100_reviewer_2_report_original_submission.pdf]

Reviewer Report

Title: Nanopore- and AI-empowered microbial viability inference

Version: Original Submission **Date:** 11/5/2024

Reviewer name: Jakob Wirbel

Reviewer Comments to Author:

Summary: Urel and colleagues present a novel computational method to predict viability from metagenomic sequencing data, using the Nanopore squiggle as input. The manuscript is well-written and present an interesting new application, bolstered in particular by the application of explainable AI. However, I have some concerns regarding the generalizability of their method, detailed below.
Major:
The way the authors try to exclude contamination in their C. abortus experiment is not optimal, since contaminatants might be at low abundance and therefore not assemble well (especially with the relatively low sequencing output overall). Instead, it would be better to map reads against the reference genome for C. abortus and check if reads predicted to be viable map or if they are unmapped in this test. Maybe viable reads instead map against a database of known contaminants, like skin-resident microbes or other known kit contaminants. (This could potentially bolster their model performance)
The authors claim that their method generalizes well from E. coli to C. abortus, which were killed in two different ways (UV and heat shock). However, if I understood correctly, their extracted DNA was left in the lab for 5 days. During this time, could exposure to sunlight over time have led to similar chemical reactions (meaning twists/kinks in the DNA as well as pyrmidine dimers)? This might be a point to discuss or it could be easily tested by incubating the DNA of the heat-killed C. abortus in the dark.
What is the time-frame of DNA degradation in which the model works best? The authors left the DNA for 5 days, but metagenomic samples are usually processed quite quickly. How would the model perform on samples that were only kept for 1 day after initial killing? At which time of incubation does the model not generalize anymore? For a potential application, it might be useful to know if DNA is viable or not, even if the cells died relatively recently (and in the dark).
Code availability: The github looks great, but as a potential user of their method, I would not want to train my own model. Is it possible to host the model, maybe on Zenodo, so that it could be more useful as an application?
Minor:
Lines 96-100 read a bit like a Nanopore commercial and are not really relevant for this paper
Line 182: shouldn't heat shock at 120 C inactivate enzymes?
Line 206: it is curious to keep the default cutoff just because the results are fine. Why not optimize the F1 score, for example? Fig1B seems to indicate that a probability threshold of 0.48 or something would give a higher F1 score. The decision to keep the threshold at the default value seems arbitrary
Line 275: interesting hypothesis. Did you observe quicker decay of pore viability in the dead versus the alive run? Could you provide the pore scan information over the time of the sequencing run as a

supplement, maybe, to back up this hypothesis?
Line 311: the number does not match the one in the table
Line 331: the dead reads are very short. Could you compare just the length of the reads with the viability predictions? Are shorter reads more likely to be predicted to be non-viable?
Fig 3a: what does normalized count mean? How about a standard histogram or density plot?
Line 442: The most recent version of dorado is v0.8.2.; did you mean v0.4.2? Please adjust.

Methods

Are the methods appropriate to the aims of the study, are they well described, and are necessary controls included? Choose an item.

Conclusions

Are the conclusions adequately supported by the data shown? Choose an item.

Reporting Standards

Does the manuscript adhere to the journal's guidelines on minimum standards of reporting? Choose an item.

Choose an item.

Statistics

Are you able to assess all statistics in the manuscript, including the appropriateness of statistical tests used? Choose an item.

Quality of Written English

Please indicate the quality of language in the manuscript: Choose an item.

Declaration of Competing Interests

Please complete a declaration of competing interests, considering the following questions:

- Have you in the past five years received reimbursements, fees, funding, or salary from an organisation that may in any way gain or lose financially from the publication of this manuscript, either now or in the future?
- Do you hold any stocks or shares in an organisation that may in any way gain or lose financially from the publication of this manuscript, either now or in the future?
- Do you hold or are you currently applying for any patents relating to the content of the manuscript?

- Have you received reimbursements, fees, funding, or salary from an organization that holds or has applied for patents relating to the content of the manuscript?
- Do you have any other financial competing interests?
- Do you have any non-financial competing interests in relation to this paper?

If you can answer no to all of the above, write 'I declare that I have no competing interests' below. If your reply is yes to any, please give details below.

I declare that I have no competing interests

I agree to the open peer review policy of the journal. I understand that my name will be included on my report to the authors and, if the manuscript is accepted for publication, my named report including any attachments I upload will be posted on the website along with the authors' responses. I agree for my report to be made available under an Open Access Creative Commons CC-BY license (http://creativecommons.org/licenses/by/4.0/). I understand that any comments which I do not wish to be included in my named report can be included as confidential comments to the editors, which will not be published.

Choose an item.

To further support our reviewers, we have joined with Publons, where you can gain additional credit to further highlight your hard work (see: https://publons.com/journal/530/gigascience). On publication of this paper, your review will be automatically added to Publons, you can then choose whether or not to claim your Publons credit. I understand this statement.

Yes Choose an item.
