# Supplementary material for: Nanopore- and AI-empowered microbial viability inference
Source: Gigascience. 2025 Sep 3;14:giaf100. doi: 10.1093/gigascience/giaf100 (PMC12405693; doi:10.1093/gigascience/giaf100)
Supplement: giaf100_Authors_Response_To_Reviewer_Comments [file giaf100_authors_response_to_reviewer_comments.pdf]

Author's Response To Reviewer Comments

Dear Hans, dear GigaScience Editorial Team, We have addressed all the specific requirements, and have added a few additional minor changes, which are all highlighted in yellow in the manuscript version with the tracked changes. Please let us know if there is anything else we should adjust. Thank you for all your (and the curators') support on the way! Best, Lara
